# The Role of Foot-Loading Factors and Their Associations with Ulcer Development and Ulcer Healing in People with Diabetes: A Systematic Review

**DOI:** 10.3390/jcm9113591

**Published:** 2020-11-07

**Authors:** Chantal M. Hulshof, Jaap J. van Netten, Mirjam Pijnappels, Sicco A. Bus

**Affiliations:** 1 Department of Rehabilitation Medicine, Amsterdam Movement Sciences, University of Amsterdam, Amsterdam UMC, Meibergdreef 9, 1105 AZ Amsterdam, The Netherlands; s.a.bus@amsterdamumc.nl; 2 Department of Human Movement Sciences, Amsterdam Movement Sciences, Vrije Universiteit Amsterdam, van der Boechorststraat 7, 1081 BT Amsterdam, The Netherlands; m.pijnappels@vu.nl

**Keywords:** diabetic foot, biomechanics, foot loading, cumulative plantar tissue stress, adherence, weight-bearing activity

## Abstract

We aimed to comprehensively and systematically review studies associating key foot-loading factors (i.e., plantar pressure, weight-bearing activity, adherence or a combination thereof) with ulcer development and ulcer healing in people with diabetes. A systematic literature search was performed in PubMed and EMBASE. We included studies if barefoot or in-shoe plantar pressure, weight-bearing activity or footwear or device adherence was measured and associated with either ulcer development or ulcer healing in people with diabetes. Out of 1954 records, 36 studies were included and qualitatively analyzed. We found low to moderate quality evidence that lower barefoot plantar pressure and higher footwear and device adherence associate with lower risk of ulcer development and shorter healing times. For the other foot-loading factors, we found low quality evidence with limited or contradictory results. For combined measures of foot-loading factors, we found low quality evidence suggesting that lower cumulative plantar tissue stress is associated with lower risk of ulcer development and higher ulcer healing incidence. We conclude that evidence for barefoot plantar pressure and adherence in association with ulcer outcome is present, but is limited for the other foot-loading factors. More comprehensive investigation in particularly the combination of foot-loading factors may improve the evidence and targeting preventative treatment.

## 1. Introduction

Foot ulcers are a common complication of diabetes mellitus, with a lifetime incidence of 19% to 34% among people with diabetes [1]. Worldwide, 18.6 million people live with a diabetic foot ulcer [2]. Once an ulcer is healed, 40% of the people develop a recurrent foot ulcer within 1 year, and this is 65% within 5 years [1]. Diabetic foot ulcers increase the risk of infection, hospitalization, amputation, mortality and result in high treatment costs [1,3,4,5]. To reduce these severe outcomes, better prevention and faster healing of diabetic foot ulcers is needed.

During weight-bearing activity, foot-to-floor contact takes place continuously, which causes repetitive loading on the foot [6,7]. High mechanical loads on the foot leads to ulcer development and delays ulcer healing, and this load needs to be reduced for ulcer prevention and ulcer healing [7,8,9]. Devices such as knee-high casts, walkers or custom-made footwear to reduce foot loading are therefore a key component in preventative and curative diabetic foot treatment, as they are primarily developed to reduce plantar pressure [8,9]. Offloading devices reduce peak plantar pressure with 14–76% compared to barefoot plantar pressure [10], which means that adherence to wearing these devices is an important contributor to reducing foot loading [9]. Thus, plantar pressure, weight-bearing activity and adherence all play a role in foot loading [7,8,9].

The combined contribution of weight-bearing activity, plantar pressure and adherence to foot loading can be expressed as the cumulative plantar tissue stress [9]. This term was introduced in 2003 by Maluf and Mueller [11], but its association with ulcer development and healing has hardly been studied [12,13]. In total, two recent narrative reviews summarized some of the studies on foot loading and ulcer development and healing. Lazzarini and colleagues [9] highlighted the significance of cumulative plantar tissue stress in ulcer development and healing, but did not systematically study its association. Chatwin and colleagues [7] emphasized the role of plantar pressure in ulcer prevention and ulcer healing, but did not focus on weight-bearing activity and adherence. Despite a variety of studies on foot-loading factors as potential determinants for either or both ulcer development and healing, a comprehensive overview of all studies that associated either one of the foot-loading factors or a combination of two or three thereof in a cumulative plantar tissue stress parameter with ulcer development and healing is lacking.

Grading of evidence and identification of knowledge gaps can help to better understand how foot-loading factors influence ulcer development and healing and therefore facilitate prevention and treatment. Our aim was to systematically review the peer-reviewed literature on the association between plantar pressure, weight-bearing activity and adherence, or a combination of those factors, and foot ulcer development and foot ulcer healing in people with diabetes.

## 2. Methods

We performed a systematic review according to the Preferred Reporting Items for Systematic Reviews and Meta-Analyses (PRISMA) statement [14]. The systematic review was prospectively registered in PROSPERO on 18 March 2020 (CRD42020170945).

### 2.1. Population Characteristics

The populations of interest for our systematic review were people either at risk of developing a diabetic foot ulcer or with a diabetic foot ulcer. For the population at risk this included people with diabetes mellitus type 1 or 2 with or without foot ulcer history [15]. We included both people with and without a history of a foot ulcer, because both give an important insight into the association between foot-loading factors and clinical outcomes.

### 2.2. Factors

We included 3 types of foot-loading factors in our systematic review, which could be studied separately or combined:

1. Plantar pressure, consisting of objectively measured barefoot and in-shoe plantar pressure measurements, usually represented as peak plantar pressure or pressure-time integral [16].
2. Weight-bearing activity, consisting of objective measurements of weight-bearing activities in daily life, and expressed as daily steps or strides, bouts of activity, duration of bouts and intensity of daily activities.
3. Adherence to wearing an offloading modality for ulcer prevention or treatment (e.g., custom-made footwear, walker or cast), assessed either objectively with measurement systems or subjectively via self-report (e.g., logbook or questionnaire), and expressed as percentage or hours of weight-bearing activity duration or of number of steps.

### 2.3. Outcomes

Primary outcomes were development of a (recurrent) foot ulcer and healing of a foot ulcer. Secondary outcomes were time to ulcer development, time to ulcer healing and wound size reduction in 4 weeks. We defined a foot ulcer in line with international guidelines as: “A break of the skin of the foot that involves as a minimum the epidermis and part of the dermis” [16]. We defined healing as: “Intact skin, meaning complete epithelialization without any drainage of a previous foot ulcer site” [16]. Despite differences in care pathways related to ulcer prevention or ulcer treatment, we included both studies on ulcer development and ulcer healing, because the foot loading factors and their working mechanisms are similar. Including them both will show their similarities and their differences, which will facilitate researchers working in both ulcer prevention and ulcer healing.

### 2.4. Eligibility Criteria

We included studies that reported findings for the population of interest, at least one of the predefined factors and one of the primary outcomes, and that reported either a direct or indirect association between factor and outcome. We defined a direct association as an association between factor and outcome that was directly statistically tested. We defined an indirect association as a description of both the factor and outcome for 2 or more groups, but where groups were classified based on another factor (e.g., treatment) and where there was no statistical testing of the association between foot-loading factors and outcome. While studies with an indirect association do not assess the direct association between factor and outcome, they give insight in the direction of the associations in different groups. Therefore, these studies contribute to the comprehensiveness of our systematic review. We included the following study designs: meta-analyses, (non)randomized controlled trials, case-control studies, cohort studies, (controlled) before-and-after studies, interrupted time series, prospective and retrospective noncontrolled studies and case series; we excluded cross-sectional studies and case reports.

### 2.5. Search Strategy

We created validation sets of approximately 10 studies per factor (Supplementary file S1) that were key studies known to the authors and that fit the scope of our systematic review, in order to validate our search strategy for completeness. Each study in the validation set had to be identified in the literature search, which was checked during development of the search strings. The final search strings (Supplementary file S2) were used to search two databases (PubMed and Excerpta Medica Database (EMBASE) via Ovid SP) on 18 March 2020, without any date and language restriction.

### 2.6. Eligibility Assessment

We used the web application Rayyan for assessing eligibility [17]. A total of 2 reviewers (C.M.H. and J.J.v.N.) independently screened the title and abstract of 200 records for eligibility based on 4 criteria: population, study design, factors and primary outcomes. If either reviewer scored the record as potentially eligible, the record was included for the full-text screening. Cohen’s kappa was calculated for agreement between reviewers. If kappa was > 0.6, the remaining records were screened by 1 reviewer, if kappa was < 0.6, all records were screened by 2 reviewers. Cohen’s kappa was 0.74 for the first 200 records, therefore the remaining records were screened by 1 reviewer (C.M.H.).

In total, 2 reviewers (C.M.H. and J.J.v.N.) independently screened 20 records of the full-text papers for final inclusion based on the aforementioned 4 criteria. Cohen’s kappa was again calculated for agreement between reviewers, and because Cohen’s kappa was 0.87, the remaining records were screened for eligibility by 1 reviewer (C.M.H.).

### 2.7. Risk of Bias Assessment

Included studies that investigated a direct association between factor and outcome were assessed by 1 reviewer (C.M.H.) for risk of bias using the Quality In Prognosis Studies (QUIPS) [18] tool, the 21-item score for reporting standards of studies on the prevention and management of foot ulcers in diabetes [19], and the Scottish Intercollegiate Guidelines Network (SIGN) critical appraisal checklists for systematic reviews and meta-analyses (https://www.sign.ac.uk/). All assessment scores were checked by a second reviewer (J.J.v.N.). Disagreements regarding risk of bias between reviewers were discussed and resolved with consensus reached. If multiple publications were available from one original study, we assessed risk of bias based on all included publications of the original study.

QUIPS tool consists of 6 different domains to score risk of bias: (1) study participation, (2) study attrition, (3) prognostic factor measurement, (4) outcome measurement, (5) study confounding and (6) statistical analysis and reporting. All domains consist of multiple items to consider for judging the overall rating per domain and the number of items vary between domains. With no validated cut-off score available for the summary score per QUIPS domain, we scored risk of bias as low when most or all items were scored as ‘yes’, and as high when all or most were scored as ‘no’ or ‘unsure’. All items and summary domain scores were discussed between the 2 reviewers until agreement was reached. Developers of QUIPS recommend against an overall score for risk of bias, therefore we scored risk of bias per domain [18]. Using the 21-item score [19], we assessed 17 items and excluded item 3, 6, 7 and 14, because these questions are only relevant for intervention studies, not for prognostic studies. All items scored as a ‘plus’ were counted as 1 and summated to calculate the total score out of 17. The 21-item score has no cut-off point for low, moderate and high risk of bias.

### 2.8. Data Extraction

Data of studies reporting a direct association between factor and outcome were extracted from all included publications by 1 reviewer (C.M.H.) and synthesized in an evidence table (including study design and setting, population characteristics, risk of bias assessment, factor outcomes and primary outcomes). All extracted data were checked by a second reviewer (J.J.v.N.). Disagreements between reviewers were discussed and resolved based on consensus. We contacted authors of publications of studies for additional information if data were available according to the methods section but not reported in the publication.

### 2.9. Evidence Statement

Finally, 1 reviewer (C.M.H.) drew conclusions based on the strength of the available evidence, formulated as evidence statements and accompanying assessment of the quality of the evidence (QoE), according to GRADE [20]. The evidence statements and QoE were checked by all coauthors (J.J.v.N., S.A.B. and M.P.). Disagreements between reviewers were discussed until consensus was reached. The reviewers rated the QoE for each formulated evidence statement as “high”, “moderate” or “low”. GRADE defines “high” as “further research is unlikely to change our confidence in our evidence statement”; “moderate” as “further research is likely to have an impact on our confidence in our evidence statement”; and “low” as “further research is very likely to have an impact on our confidence in our evidence statement” [20]. The rating was determined based on the risk of bias, consistency of results, publication bias and effect size [20].

## 3. Results

**Figure 1:**
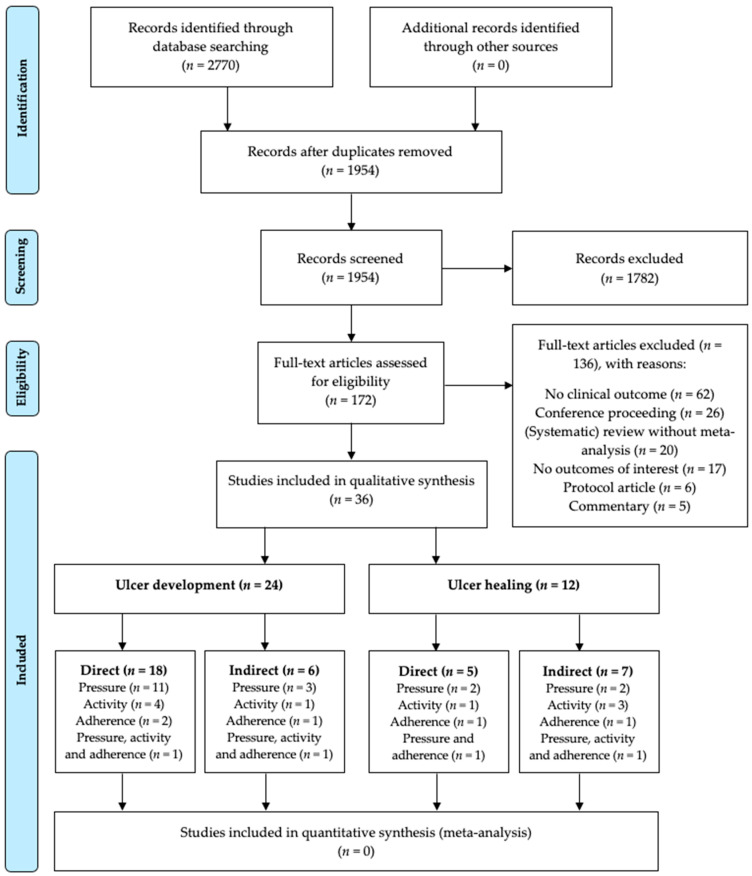
PRISMA flow diagram.

**Figure 2:**
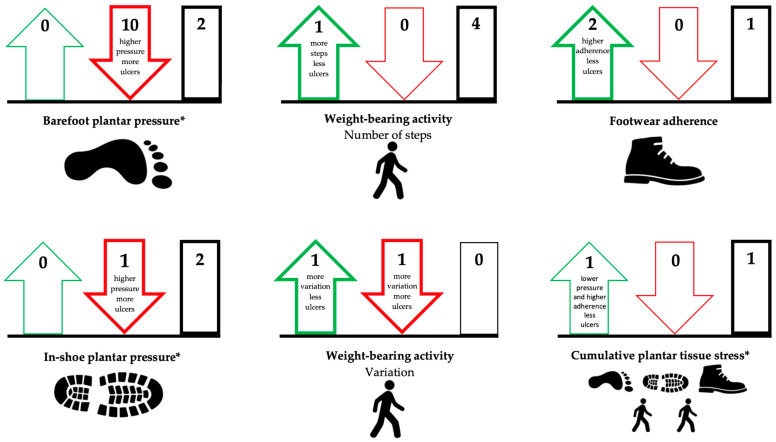
Overview of the results per foot-loading factor for ulcer development. Arrow up (**green**) = favorable association; arrow down (**red**) = unfavorable association; rectangle (**black**) = no association. Thick outline of arrow or rectangle means that there is evidence, thin outline means that there is no evidence. The number at the top in the arrow or rectangle is the number of studies found for the association in that direction. * Some publications investigated multiple associations, therefore the numbers in the figure do not always correspond to the number of included publications.

**Figure 3:**
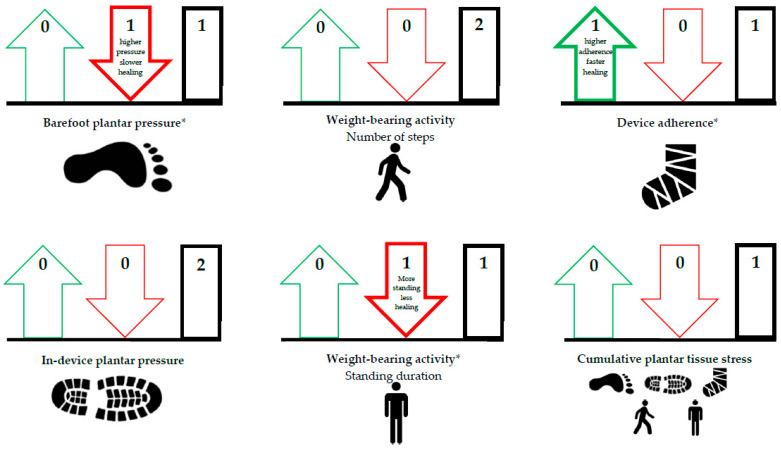
Overview of the results per foot-loading factor for ulcer healing. Arrow up (**green**) = favorable association; arrow down (**red**) = unfavorable association; rectangle (**black**) = no association. Thick outline of arrow or rectangle means that there is evidence, thin outline means that there is no evidence. The number at the top in the arrow or rectangle is the number of studies found for the association in that direction. * Some publications investigated multiple associations, therefore the numbers in the figure do not always correspond to the number of included publications.

In total, 36 publications were included in our systematic review (see for details the PRISMA flow diagram in Figure 1). We will describe the results with the evidence statements for each foot-loading factor and combination thereof, separately for the outcomes ulcer development and ulcer healing (Table 1). A schematic overview of the results is depicted in Figure 2 and Figure 3. Risk of bias was assessed for the 23 publications that investigated a direct association between factor and outcome (Table 2, see for details Tables S1–S3). Taken together per domain of QUIPS, study attrition and prognostic factor measurement were mostly scored as low risk of bias, statistical analysis and reporting as moderate risk of bias, study participation and outcome measurement equally frequent as low, moderate or high risk of bias, and study confounding as high risk of bias.

### 3.1. Ulcer Development

#### 3.1.1. Plantar Pressure

For barefoot plantar pressure, we included 11 publications investigating a direct association with ulcer development (six prospective cohorts, two randomized controlled trials, one retrospective cohort, one before and after study, one meta-analysis). Pham et al. [21], Lavery et al. [23], Grimm et al. [25], Kästenbauer et al. [24], Ulbrecht et al. [26], Deschamps et al. [27] and Waaijman et al. [12] (combined number of participants (*n*), *n* = 2463, combined number of events (e), e = 446) found that higher barefoot peak plantar pressure was associated with increased risk of ulcer development, Crawford et al. [30] confirmed this in a meta-analysis based on two of these studies (see for details Table S4). Murray et al. [28] and Qiu et al. [29] (*n* = 128, e = 8) found no association between barefoot peak plantar pressure and risk of ulcer development. Furthermore, Pham et al. [21], Caselli et al. [22], Lavery et al. [23] and Murray et al. [28] (*n* = 1977, e = 342) found that a cut-off for barefoot peak pressure between 588 and 981 kPa was associated with increased risk of ulcer development. Veves et al. [42] (*n* = 86, e = 15) showed in a study with an indirect association that barefoot peak plantar pressure of >1206 kPa was predictive for foot ulcer development. These results suggest that higher barefoot plantar pressure is associated with increased risk of ulcer development (QoE: moderate) (Table 1; Figure 2).

For in-shoe plantar pressure, we included two publications investigating a direct association with ulcer development (one prospective cohort, one randomized controlled trial). In a detailed analysis of a randomized controlled trial of Bus et al. [43], Waaijman et al. [12] (*n* = 171, e = 71) found higher in-shoe peak plantar pressure (measured quarterly) to be associated with increased risk of plantar ulcers from unrecognized trauma, but not when any plantar foot ulcer was used as outcome (see for details Table S4). Ledoux et al. [31] (*n* = 591, e = 47) found no association between in-shoe peak plantar pressure (measured once) and risk of ulcer development. Viswanathan et al. [44] (*n* = 241, e = 23) showed in a study with an indirect association that peak plantar pressure in therapeutic footwear decreased over time, while peak plantar pressure in non-therapeutic footwear increased over time, and non-therapeutic footwear increased the risk of ulcer development compared to therapeutic footwear. Fernandez et al. [45] (*n* = 100, e = 18) showed in a study with indirect association lower in-shoe plantar pressure in therapeutic footwear and less risk of ulcer development compared to nontherapeutic footwear. These results seem to suggest that the association of in-shoe plantar pressure with risk of ulcer development is unclear (QoE: low) (Table 1; Figure 2).

#### 3.1.2. Weight-Bearing Activity

For weight-bearing activity, we included five publications investigating a direct association with ulcer development (two prospective cohorts, two randomized controlled trials, one prospective mixed method). Lemaster et al. [33], Waaijman et al. [12], Mueller et al. [34] and Schneider et al. [35] (*n* = 602, e = 131) found no association between level of weight-bearing activity (number of steps) and risk of ulcer development (see for details Table S5). Armstrong et al. [32] (*n* = 100, e = 8) found that a lower average daily activity level was associated with increased risk of ulcer development. Results on the effect of variation in weight-bearing activity were contradictory between studies: Armstrong et al. [32] found that more variation in weight-bearing activity was associated with increased risk of ulcer development, while Waaijman et al. [12] (*n* = 171, e = 71) found that less variation in steps per day was associated with increased risk of ulcer development. Lemaster et al. [46] (*n* = 79, e = 15) showed in a study with an indirect association that there was no difference in number of steps per day and risk of ulcer development between their weight-bearing activity intervention group and control group. These results suggest that the association of level and variation of weight-bearing activity with risk of ulcer development is unclear (QoE: low) (Table 1; Figure 2).

#### 3.1.3. Footwear Adherence

For footwear adherence, we included three publications investigating a direct association with ulcer development (one prospective cohort, one retrospective cohort, one randomized controlled trial). Chantelau et al. [36] and Connor et al. [37] (*n* = 134) found that higher footwear adherence (assessed subjectively) was associated with lower risk of ulcer development (see for details Table S6). Waaijman et al. [12] (*n* = 171, e = 71) found no direct association between footwear adherence (measured objectively) and risk of ulcer development. Lavery et al. [47] (*n* = 299, e = 13) found in a study with an indirect association no difference in footwear adherence between participants who received shear-reducing insoles and those who received standard care; however, ulcers developed more frequently in people with standard care compared to shear-reducing insoles. These results seem to suggest that lower footwear adherence is associated with increased risk of ulcer development (QoE: low) (Table 1; Figure 2).

#### 3.1.4. Cumulative Plantar Tissue Stress

For cumulative plantar tissue stress, we included one publication investigating a direct association with ulcer development (one randomized controlled trial). Waaijman et al. [12] (*n* = 171, e = 71) combined barefoot and in-shoe plantar pressure, weight-bearing activity (i.e., number of daily steps) and footwear adherence to determine the cumulative plantar tissue stress on the foot. Cumulative plantar tissue stress was higher in people who developed a recurrent plantar ulcer (715 ± 538 MPa⋅s/day) or a recurrent ulcer from unrecognized trauma (423 ± 292 MPa⋅s/day) compared to people who did not develop a plantar ulcer (652 ± 436 MPa⋅s/day) or an ulcer from unrecognized trauma (361 ± 279 MPa⋅s/day), respectively (see for details Table S7). However, the associations between cumulative plantar tissue stress and risk of ulcer development were not statistically significant (*p* = 0.453 and *p* = 0.162, respectively). The percentage of people with an in-shoe peak plantar pressure of <200 kPa and footwear adherence >80% was lower in those who developed a recurrent plantar ulcer (9.0%) or a recurrent ulcer from unrecognized trauma (17.9%) compared to people who did not develop a plantar ulcer (10.2%) or an ulcer from unrecognized trauma (27.3%), respectively. These associations were statistically significant (*p* = 0.012 and *p* = 0.019, respectively) [12]. These results seem to suggest that higher cumulative plantar tissue stress increases the risk of ulcer development (QoE: low) (Table 1; Figure 2).

### 3.2. Ulcer Healing

#### 3.2.1. Plantar Pressure

For barefoot plantar pressure, we included one publication investigating a direct association with ulcer healing (one prospective cohort). Armstrong et al. [38] (*n* = 25, e = 25) found that lower barefoot peak plantar pressure was associated with shorter ulcer healing times and they also showed that a cut-off of <990 kPa was nearly associated (*p* = 0.05) with shorter ulcer healing times (see for details Table S8). Patel et al. [48] (*n* = 16, e = 16) showed in a study with an indirect association that participants with chronic foot ulcers (i.e., 36 (6–64) weeks present at surgery) who underwent a metatarsal head resection had lower peak plantar pressures at the ulcer location postoperatively compared to preoperatively and all patients healed after 8 ± 2 weeks post-resection. These results seem to suggest that lower barefoot plantar pressure is associated with shorter ulcer healing times (QoE: low) (Table 1; Figure 3).

For in-device plantar pressure, we included two publications investigating a direct association with ulcer healing (two prospective cohorts). In a detailed analysis of a randomized controlled trial, Bus et al. [49] and van Netten et al. [13] (in removable knee-high and ankle-high devices) (*n* = 31, e = 21) (in nonremovable ankle-high devices) found that measured in-device peak plantar pressure and pressure time integral were not associated with ulcer healing incidence (see for details Table S8). Jarl et al. [39] (*n* = 7, e = 7) (in nonremovable ankle-high devices) found that measured in-device peak plantar pressure was not associated with ulcer healing times. Gutekunst et al. [50] (*n* = 23, e = 14) showed in a study with an indirect association that there was no difference in peak plantar pressures between wearing a non-removable and removable device, but ulcer healing incidence was higher in a nonremovable device compared to a removable device. These results seem to suggest that in-device plantar pressure in already adequately offloading devices are not associated with ulcer healing incidence (QoE: low) (Table 1; Figure 3).

#### 3.2.2. Weight-Bearing Activity

For weight-bearing activity, we included two publications investigating a direct association with ulcer healing (one prospective cohort, one randomized controlled trial). Van Netten et al. [13] and Najafi et al. [40] (*n* = 80, e = 43) found no association between the number of steps per day taken and ulcer healing incidence at any time point (see for details Table S9). However, Najafi et al. [40] (*n* = 49, e = 22) found an association with shorter duration of standing at last visit leading to an increased ulcer healing incidence, while duration of standing at baseline was not associated with ulcer healing. Armstrong et al. [51] and Lavery et al. [52] (*n* = 196, e = 76) found in their studies with an indirect association a lower number of steps per day and a higher ulcer healing incidence in nonremovable devices compared to removable devices. Saltzman et al. [53] (*n* = 40, e = 32) found in a study with indirect association that number of steps per day did not significantly affect healing incidence in nonremovable devices [53]. These results seem to suggest that the association of the level of weight-bearing activity with ulcer healing incidence is unclear (QoE: low) (Table 1; Figure 3).

#### 3.2.3. Device Adherence

For device adherence, we included one publication investigating a direct association with ulcer healing (one prospective cohort). Crews et al. [41] (*n* = 79, e = 19) found that higher adherence to wearing a removable offloading walker was associated with shorter ulcer healing times at 6 weeks. However, they found no association between device adherence and ulcer healing incidence at 6 weeks (see for details Table S10). Ha Van et al [54] (*n* = 93, e = 70) showed in a study with an indirect association that both device adherence and incidence of ulcer healing were better in a nonremovable cast compared to a removable offloading shoe. These results seem to suggest that higher device adherence is associated with shorter ulcer healing times (QoE: low) (Table 1; Figure 3).

#### 3.2.4. Cumulative Plantar Tissue Stress

For cumulative plantar tissue stress, we included one publication investigating a direct association with ulcer healing (one prospective cohort). Van Netten et al. [13] (*n* = 31, e = 21) combined in-device plantar pressure and weight-bearing activity (i.e., number of daily steps) to determine a cumulative plantar tissue stress index. Cumulative plantar tissue stress was lower in people who healed from a plantar ulcer (155 ± 131 MPa⋅s/day) compared to those who did not heal from a plantar ulcer (207 ± 215 MPa⋅s/day) (see for details Table S11). Among people who self-reported to be adherent to wearing their devices, cumulative plantar tissue stress was lower in people who had ≥75% ulcer area reduction at 4 weeks compared to those who had <75% ulcer are reduction at 4 weeks (140 ± 137 and 275 ± 209 MPa⋅s/day, respectively) [13]. However, both associations were not statistically significant (*p* = 0.71 and *p* = 0.09). These results seem to suggest that lower cumulative plantar tissue stress increases the chance of ulcer healing (QoE: low) (Table 1; Figure 3).

## 4. Discussion

We systematically reviewed the peer-reviewed literature for studies with either plantar pressure, weight-bearing activity and footwear or device adherence or a combination of those foot-loading factors, and their associations with ulcer development and ulcer healing in people with diabetes as outcomes. For single foot-loading factors, we found low to moderate quality evidence that higher barefoot plantar pressure is associated with increased risk of ulcer development and longer ulcer healing times. Furthermore, we found low quality evidence that seems to suggest that higher footwear adherence and device adherence is associated with lower risk of ulcer development and shorter healing times. For the other foot-loading factors (i.e., in-shoe and in-device plantar pressure, weight-bearing activity and cumulative plantar tissue stress), we found low quality evidence with few or contradictory results. Taken together, this is the first systematic overview including multiple key foot-loading factors and a combination thereof, and shows both the available evidence as well as clear knowledge gaps for which research is urgently needed.

### 4.1. Evidences for Underlying Foot-Loading Factors for Ulcer Outcomes

#### 4.1.1. Ulcer Development

We found sufficient evidence that associates higher barefoot plantar pressure with increased risk of ulcer development. With 11 publications and a total of 2627 participants, this was the most frequently studied association. Between studies, barefoot peak plantar pressures varied largely and a variety of pressure-threshold cut-offs were used. This may be due to the different measurement systems used between studies or differences in populations, but no threshold was validated in another study. For the association of in-shoe plantar pressure (2 studies, *n* = 762) and level of weight-bearing activity (5 studies, *n* = 702) with risk of ulcer development, we found very few studies and thus limited evidence, despite its importance in daily clinical practice. The two studies (*n* = 271) reporting on the association between variation in weight-bearing activity and risk of ulcer development showed contradictory findings, demonstrating that more studies are needed on this association. We found some evidence that a higher footwear adherence (3 studies, *n* = 305) is associated with reduced risk of ulcer development, but there are still areas in need of investigation, especially because in all but one study evidence came from subjective measurements of device adherence.

#### 4.1.2. Ulcer Healing

Overall, we found fewer studies on ulcer healing than on ulcer development, with markedly fewer participants included. We found evidence that higher barefoot plantar pressure (1 study, *n* = 25) was associated with shorter ulcer healing times, but we found no evidence for the association of barefoot plantar pressure with ulcer healing incidence. For the association of in-device plantar pressure (2 studies, *n* = 38) and number of steps (2 studies, *n* = 80) with ulcer healing outcomes we found no evidence, but we found evidence from one study (*n* = 49) that shorter duration of standing at last visit was associated with increased ulcer healing incidence. We found evidence (1 study, *n* = 79) that higher device adherence was associated with shorter ulcer healing times, but no evidence for the association between device adherence and ulcer healing incidence. With the limited number of studies and participants, it is clear that more investigation on the associations between foot-loading factors and ulcer healing is needed.

#### 4.1.3. Limitations in Associations Found

Limitations in studying these associations exist due to the limited number of studies initially conducted to assess the association between foot-loading factors and either ulcer development or healing, while this is of particular interest to better understand why ulcers develop or heal. Furthermore, large differences between studies in populations and settings included may explain the less clear associations found. The limitation of having only limited studies applies especially for associations with ulcer healing.

The strong evidence from many randomized controlled trials that pressure-reducing devices (i.e., custom-made footwear and knee-high offloading devices) are effective in ulcer prevention and ulcer healing [55,56,57] suggests a clear association between high in-shoe or in-device pressure and poor ulcer outcomes. However, most of these clinical studies did not investigate foot-loading factors, and could therefore not be included in our review. In those studies that did investigate foot-loading factors, particularly the two small studies that measured in-device plantar pressures in association with ulcer healing [13,39], all participants were provided with adequate offloading devices, hence variation in pressure was small. This restricts the ability to find associations between in-device pressure and ulcer healing. Another limitation is that the majority of the studies did not include the other foot-loading factors, while these foot-loading factors interact and may therefore hide associations. To improve understanding of ulcer outcomes with these offloading interventions, the association between foot-loading factors and ulcer development and healing needs more comprehensive investigation. This will help in providing more tailored advice on, for example, the amount of weight-bearing activity possible with these offloading interventions.

### 4.2. Strengths and Limitations of Included Studies

Strengths of the included studies were that most of the studies reported the method in detail, increasing the reproducibility of studies. In addition, study attrition was low or described in detail in most of the studies, which reduces bias among studies.

A limitation of included studies was that foot-loading factors were almost always measured only once during follow-up. However, pressures may change over time [43,58], activity patterns vary [32,46] and the amount of device adherence is not consistent [40,59]. This may explain the few associations found. With sensors becoming smaller and easier to use [60], we recommend to repeat measurements over time or to measure continuously, so to better investigate associations with ulcer outcomes. A limitation in comparing studies was that different outcome measures were used for the same foot-loading factor. While measures were mostly consistent for barefoot plantar pressure, for weight-bearing activity some studies used undefined units, while others were a mix of daily steps, bouts of activity and time active. For device adherence, no study used the same measurement method. This limits the generalizability of findings, and we suggest future studies only use validated, quantitative and objective methods, and to report multiple outcomes. Another limitation, as shown in the risk of bias assessment, was the frequent absence of essential reporting details and limited reporting of high-quality multivariate analyses. These various weaknesses influence the quality of evidence and reduce clarity with regard to the direction of the associations found. For more representative and high-quality data on the associations between foot-loading factors and ulcer outcomes, future studies should take these weaknesses into account.

All but one of our evidence statements were based on low quality evidence. This was unfortunate given the importance of foot-loading factors in daily diabetic foot practice. It was particularly the result of having only limited studies, with frequently few participants, per association. Furthermore, there were some inconsistencies in the results, and no study scored low risk of bias for all QUIPS domains. However, this also shows that with new, high-quality, studies, the level of evidence in the field can be greatly improved.

Another limitation with the included studies was the absence of prognostic studies focusing on the association between shear stress and clinical outcomes. While shear stress is a component of foot loading and could be associated with clinical outcomes [61,62], with no longitudinal studies available we could not investigate this association in the current review.

In addition to studies reporting a direct association, we also included multiple studies where an indirect association between foot-loading factor and ulcer outcomes was reported; for example in a randomized controlled trial where both weight-bearing activity and ulcer incidence were reported for the two study arms, but without reporting directly on weight-bearing activity in those with and without an ulcer [46]. For studies that have data on foot-loading factors and ulcer outcomes available, we strongly recommend to report their associations, to improve insights from such studies.

### 4.3. Cumulative Plantar Tissue Stress

The generally low or lack of evidence found in associating single foot-loading factors with ulcer outcomes does not necessarily mean that evidence is absent. Hypothesized associations may still be present, but studies are often limited by the fact that foot loading is not measured as comprehensively as possible, creating weak associations between single factors and clinical outcomes. Furthermore, the effect modification of or interaction with other foot-loading factors (e.g., higher pressures but less weight-bearing activity) is not accounted for in the single-factor studies we found, which may explain some of the findings.

The combination of multiple foot-loading factors is expressed in the cumulative plantar tissue stress measures, which was only studied twice in association with an ulcer outcome [12,13]. While both studies found lower cumulative plantar tissue stresses in people with better clinical outcomes, this was not statistically significant. However, cumulative plantar tissue stress was measured for 1 week only during follow-up, limiting interpretation. Furthermore, in the study on ulcer healing only plantar pressure and weight-bearing activity were combined, but not device adherence, and this study had a limited number of participants. We recommend for future research to measure cumulative plantar tissue stress as combination of all three foot-loading factors in line with recent recommendations [9], and to do so for multiple weeks or continuously during follow-up.

### 4.4. Alternative Approaches on Understanding the Foot-Loading Pathways of Ulcer Outcomes

We suggest that more research is needed that investigates alternative approaches to calculate cumulative plantar tissue stress and its association with ulcer outcomes. Several aspects deserve attention. First, the currently recommended method concerns multiplying pressure-time integrals obtained during midgait steps in the laboratory with the number of steps taken in daily life outside the lab [9]. This does not account for differences in pressure between daily-life activities [63,64,65], nor differences in walking speed between the lab and real-life [66]. Secondly, the association between cumulative plantar tissue stress and ulcer outcomes may not be a linear one. Perhaps exceeding a threshold in cumulative plantar tissue stress determines if an ulcer develops or ulcer heals, and this could either be a prolonged high level of stress or an unusual variation in stress. Further, this threshold may even differ between individuals, as well as over time [67]. Thirdly, the association between tissue stress and ulcer development might follow a U-shape, rather than a linear curve or a threshold. The physical stress theory of Kluding and colleagues [68] suggests that stress on the foot can either be too high or too low, and both may lead to poor ulcer outcomes. These alternative approaches might contribute to better predicting ulcer development and ulcer healing, but this requires more research on the methods for calculating cumulative plantar tissue stress, and on its (nonlinear) association with both ulcer development and ulcer healing.

### 4.5. Methodological Reflection

Firstly, risk of bias was assessed by only one reviewer; however, a second reviewer checked all scores. We further compared our risk of bias assessments with other systematic reviews [55,69], and found consistency in assessments. Secondly, we used the 21-item score to assess risk of bias, while this score is specifically developed for intervention studies and not for prognostic studies [19]. Since this is the only score specific for studies in the field of diabetic foot disease, we used the questions of interest that apply to prognostic studies, and only used the 21-item score as an addition to the QUIPS risk of bias assessment.

We limited our systematic review to studies with a longitudinal design, thereby excluding interesting cross-sectional studies on foot-loading factors in people with diabetes (e.g. [70,71,72,73]). However, the nature of a cross-sectional study design does not allow to investigate the association of foot-loading factors with clinical outcomes. An overview of such cross-sectional studies might be useful to provide insight in characteristics of foot-loading factors in different populations, but that was outside the scope of the current systematic review.

### 4.6. Implications for Clinical Practice

The findings on barefoot pressure and device adherence confirm the importance of providing people with diabetic foot disease with pressure-reducing interventions, and ensure adherence to wearing these [15]. Further, the findings stress that to determine the loading of the foot, it is important to look beyond a single foot-loading factor, as single foot-loading factors are likely insufficient to understand treatment progress. Clinicians should gain understanding of the plantar pressures, weight-bearing activities, and device adherence of the patient. To provide more accurate clinical advice about the cumulative stress on the foot, we need more research and insight in those foot-loading factors and their interaction, in association with ulcer development and ulcer healing.

## 5. Conclusions

We showed evidence for barefoot plantar pressure and adherence in association with ulcer development and healing, but not sufficient evidence for in-shoe and in-device plantar pressure and weight-bearing activity in people with diabetes. More comprehensive and prospective research, including an adequate number of participants, investigating the combination of foot-loading factors and their interaction is needed. With higher quality evidence, we can better understand the clinical outcomes, and improve targeting treatment for foot ulcer prevention and healing for people with diabetes.

## Figures and Tables

**Table 1:** Evidence statements and quality of evidence (QoE) for the associations between foot-loading factors and ulcer development and ulcer healing.

Outcome	Factor	Evidence Statement	QoE	References
Ulcer development	Barefoot plantar pressure	Higher barefoot plantar pressure is associated with increased risk of ulcer development.	Moderate	Pham et al. [21], Caselli et al. [22], Lavery et al. [23], Kästenbauer et al. [24], Grimm et al. [25], Ulbrecht et al. [26], Deschamps et al. [27], Murray et al. [28], Qiu et al. [29], Waaijman et al. [12], Crawford et al. [30]
	In-shoe plantar pressure	The association of in-shoe plantar pressure with risk of ulcer development seems unclear.	Low	Waaijman et al. [12], Ledoux et al. [31]
	Weight-bearing activity	The association of level and variation of weight-bearing activity with risk of ulcer development is unclear.	Low	Armstrong et al. [32], Lemaster et al. [33], Waaijman et al. [12], Mueller et al. [34], Schneider et al. [35]
	Footwear adherence	Lower footwear adherence seems associated with increased risk of ulcer development.	Low	Chantelau et al. [36], Connor et al. [37], Waaijman et al. [12]
	Cumulative plantar tissue stress	Higher cumulative plantar tissue stress seems to increase the risk of ulcer development.	Low	Waaijman et al. [12]
Ulcer healing	Barefoot plantar pressure	Lower barefoot plantar pressure seems associated with shorter ulcer healing times.	Low	Armstrong et al. [38]
	In-device plantar pressure	In-device plantar pressure in already adequately offloading devices seems not associated with ulcer healing incidence and times.	Low	Van Netten et al. [13], Jarl et al. [39]
	Weight-bearing activity	The association of level of weight-bearing activity with ulcer healing incidence seems unclear.	Low	Van Netten et al. [13], Najafi et al. [40]
	Device adherence	Higher device adherence seems associated with shorter ulcer healing times.	Low	Crews et al. [41]
	Cumulative plantar tissue stress	Lower cumulative plantar tissue stress seems to increase the chance of ulcer healing.	Low	Van Netten et al. [13]

**Table 2:** QUIPS risk of bias score per foot-loading factor and primary outcome.

		Total	Ulcer Development					Ulcer Healing				
		(*n* = 20) *	Barefoot Plantar Pressure (*n* = 8)	In-Shoe Plantar Pressure (*n* = 2)	Weight-Bearing Activity (*n* = 5)	Footwear Adherence (*n* = 3)	Cumulative Plantar Tissue Stress (*n* = 1)	Barefoot Plantar Pressure (*n* = 1)	In-Device Plantar Pressure (*n* = 2)	Weight-Bearing Activity (*n* = 2)	Device Adherence (*n* = 1)	Cumulative Plantar Tissue Stress (*n* = 1)
Study participation	L	7	4	2	2	1	1		1	1		1
	M	6	3		1	1					1	
	H	7	1		2	1		1	1	1		
Study attrition	L	14	6		3	1		1	2	1	1	1
	M	4	1	2	2	1	1			1		
	H	2	1			1						
Prognostic factor measurement	L	10	5		2			1	1	1	1	1
	M	9	3	2	3	2	1		1	1		
	H	1				1						
Outcome measurement	L	5	2	1	3	1	1			1		
	M	7	3	1					2	1	1	1
	H	8	3		2	2		1				
Study confounding	L	5	2	2	2	1	1	1				
	M	5	3						1	1	1	1
	H	10	3		3	2			1	1		
Statistical analysis and reporting	L	5	2	2	2	1	1	1				
	M	12	5		3	2			1	1	1	1
	H	3	1						1	1		

QUIPS: Quality In Prognosis Studies; L = low (green), M = moderate (orange) and H = high (red) risk of bias. * 23 publications investigating a direct association were included. These were based on 21 original studies, with two studies having two publications each. Further, one study was a meta-analysis, and risk of bias could not be assessed with QUIPS; therefore, a total 20 studies are represented in this table. Some studies reported on multiple foot-loading factors, therefore the sum of all studies in a row could be more than 20, but in the total score these studies were counted only once.

## Supplementary Materials

The following are available online at https://www.mdpi.com/2077-0383/9/11/3591/s1, Table S1. Risk of bias assessment: QUIPS tool, Table S2. Risk of bias assessment: 21-item score, Table S3. Risk of bias assessment: SIGN, Table S4. Plantar pressure-ulcer development, Table S5. Weight-bearing activity-ulcer development, Table S6. Footwear adherence-ulcer development, Table S7. Cumulative plantar tissue stress-ulcer development, Table S8. Plantar pressure-ulcer healing, Table S9. Weight-bearing activity-ulcer healing, Table S10. Device adherence-ulcer healing, Table S11. Cumulative plantar tissue stress-ulcer healing, Supplementary file S1. Validation Sets, Supplementary file S2. Search Strings.
