# Supplementary material for: The Role of Foot-Loading Factors and Their Associations with Ulcer Development and Ulcer Healing in People with Diabetes: A Systematic Review
Source: J Clin Med. 2020 Nov 7;9(11):3591. doi: 10.3390/jcm9113591 (PMC7694972; doi:10.3390/jcm9113591)
Supplement: Supplementary file 1 [file jcm-09-03591-s001.zip › jcm-977657-supplementary files.pdf]

Supplementary file S1. Validation Sets.

1. Validation Set per Foot-Loading Factor

1.1. Plantar Pressure

1. Chatwin, K.E.; Abbott, C.A.; Boulton, A.J.M.; Bowling, F.L.; Reeves, N.D. The role of foot pressure measurement in the prediction and prevention of diabetic foot ulceration—A comprehensive review. *Diabetes. Metab. Res. Rev.* **2019**, *36*, 1–14, doi:10.1002/dmrr.3258.
2. Lazzarini, P.A.; Crews, R.T.; Van Netten, J.J.; Bus, S.A.; Fernando, M.E.; Chadwick, P.J.; Najafi, B. Measuring Plantar Tissue Stress in People With Diabetic Peripheral Neuropathy: A Critical Concept in Diabetic Foot Management. *J. Diabetes Sci. Technol.* **2019**, *13*, 869–880, doi:10.1177/1932296819849092.
3. Maluf, K.; Mueller, M.J. Comparison of physical activity and cumulative plantar tissue stress among subjects with and without diabetes mellitus and a history of recurrent plantar ulcers. *Clin. Biomech.* **2003**, *18*, 567–575, doi:10.1016/s0268-0033(03)00118-9.
4. Ulbrecht, J.S.; Hurley, T.; Mauger, D.T.; Cavanagh, P.R. Prevention of Recurrent Foot Ulcers With Plantar Pressure-Based In-Shoe Orthoses: The CareFUL Prevention Multicenter Randomized Controlled Trial. *Diabetes Care* **2014**, *37*, 1982–1989, doi:10.2337/dc13-2956.
5. Lavery, L.A.; Armstrong, D.G.; Wunderlich, R.P.; Tredwell, J.; Boulton, A.J. Predictive Value of Foot Pressure Assessment as Part of a Population-Based Diabetes Disease Management Program. *Diabetes Care* **2003**, *26*, 1069–1073, doi:10.2337/diacare.26.4.1069.
6. Waaijman, R.; De Haart, M.; Arts, M.L.J.; Wever, D.; Verlouw, A.J.W.E.; Nollet, F.; Bus, S.A. Risk Factors for Plantar Foot Ulcer Recurrence in Neuropathic Diabetic Patients. *Diabetes Care* **2014**, *37*, 1697–1705, doi:10.2337/dc13-2470.
7. Bus, S.A.; Van Netten, J.J.; Kottink, A.I.; A Manning, E.; Spraul, M.; Woittiez, A.-J.; Van Baal, J.G. The efficacy of removable devices to offload and heal neuropathic plantar forefoot ulcers in people with diabetes: a single-blinded multicentre randomised controlled trial. *Int. Wound J.* **2017**, *15*, 65–74, doi:10.1111/iwj.12835.
8. Veves, A.; Murray, H.J.; Young, M.J.; Boulton, A.J.M. The risk of foot ulceration in diabetic patients with high foot pressure: a prospective study. *Diabetologia* **1992**, *35*, 660–663, doi:10.1007/bf00400259.
9. Frykberg, R.G.; A Lavery, L.; Pham, H.; Harvey, C.; Harkless, L.; Veves, A. Role of Neuropathy and High Foot Pressures in Diabetic Foot Ulceration. *Diabetes Care* **1998**, *21*, 1714–1719, doi:10.2337/diacare.21.10.1714.
10. Owings, T.M.; Apelqvist, J.; Stenström, A.; Becker, M.; Bus, S.A.; Kalpen, A.; Ulbrecht, J.S.; Cavanagh, P.R. Plantar pressures in diabetic patients with foot ulcers which have remained healed. *Diabet. Med.* **2009**, *26*, 1141–1146, doi:10.1111/j.1464-5491.2009.02835.x.
11. Arts, M.L.J.; Waaijman, R.; De Haart, M.; Keukenkamp, R.; Nollet, F.; Bus, S.A. Offloading effect of therapeutic footwear in patients with diabetic neuropathy at high risk for plantar foot ulceration. *Diabet. Med.* **2012**, *29*, 1534–1541, doi:10.1111/j.1464-5491.2012.03770.x.

1.2. Weight-Bearing Activity

1. LeMaster, J.W.; Mueller, M.J.; E Reiber, G.; Mehr, D.R.; Madsen, R.W.; Conn, V.S. Effect of Weight-Bearing Activity on Foot Ulcer Incidence in People With Diabetic Peripheral Neuropathy: Feet First Randomized Controlled Trial. *Phys. Ther.* **2008**, *88*, 1385–1398, doi:10.2522/ptj.20080019.
2. Najafi, B.; Grewal, G.S.; Bharara, M.; Menzies, R.; Talal, T.K.; Armstrong, D.G. Can't Stand the Pressure: The Association Between Unprotected Standing, Walking, and Wound Healing in People With Diabetes. *J. Diabetes Sci. Technol.* **2016**, *11*, 657–667, doi:10.1177/1932296816662959.
3. Armstrong, D.G.; Lavery, L.A.; Holtz-Neiderer, K.; Mohler, M.J.; Wendel, C.S.; Nixon, B.P.; Boulton, A.J. Variability in Activity May Precede Diabetic Foot Ulceration. *Diabetes Care* **2004**, *27*, 1980–1984, doi:10.2337/diacare.27.8.1980.
4. Waaijman, R.; De Haart, M.; Arts, M.L.J.; Wever, D.; Verlouw, A.J.W.E.; Nollet, F.; Bus, S.A. Risk Factors for Plantar Foot Ulcer Recurrence in Neuropathic Diabetic Patients. *Diabetes Care* **2014**, *37*, 1697–1705, doi:10.2337/dc13-2470.
5. Van Netten, J.J.; Van Baal, J.G.; Bril, A.; Wissink, M.; Bus, S.A. An exploratory study on differences in cumulative plantar tissue stress between healing and non-healing plantar neuropathic diabetic foot ulcers.

Clin. Biomech.* **2018**, *53*, 86–92, doi:10.1016/j.clinbiomech.2018.02.012.
6. Armstrong, D.G.; Nguyen, H.C.; Lavery, L.A.; Van Schie, C.H.; Boulton, A.J.; Harkless, L.B. Off-Loading the Diabetic Foot Wound: A randomized clinical trial. *Diabetes Care* **2001**, *24*, 1019–1022, doi:10.2337/diacare.24.6.1019.
7. LeMaster, J.W.; Reiber, G.E.; Smith, D.G.; Heagerty, P.J.; Wallace, C. Daily Weight-Bearing Activity Does Not Increase the Risk of Diabetic Foot Ulcers. *Med. Sci. Sports Exerc.* **2003**, *35*, 1093–1099, doi:10.1249/01.mss.0000074459.41029.75.
8. A Lazzarini, P.; Crews, R.T.; Van Netten, J.J.; Bus, S.A.; Fernando, M.E.; Chadwick, P.J.; Najafi, B. Measuring Plantar Tissue Stress in People With Diabetic Peripheral Neuropathy: A Critical Concept in Diabetic Foot Management. *J. Diabetes Sci. Technol.* **2019**, *13*, 869–880, doi:10.1177/1932296819849092.
9. Maluf, K.; Mueller, M.J. Comparison of physical activity and cumulative plantar tissue stress among subjects with and without diabetes mellitus and a history of recurrent plantar ulcers. *Clin. Biomech.* **2003**, *18*, 567–575, doi:10.1016/s0268-0033(03)00118-9.

1.3. Adherence

1. Bus, S.A.; Waaijman, R.; Arts, M.; De Haart, M.; Busch-Westbroek, T.; Van Baal, J.; Nollet, F. Effect of Custom-Made Footwear on Foot Ulcer Recurrence in Diabetes: A multicenter randomized controlled trial. *Diabetes Care* **2013**, *36*, 4109–4116, doi:10.2337/dc13-0996.
2. Crews, R.T.; Shen, B.-J.; Campbell, L.; Lamont, P.J.; Boulton, A.J.; Peyrot, M.; Kirsner, R.S.; Vileikyte, L. Role and Determinants of Adherence to Off-loading in Diabetic Foot Ulcer Healing: A Prospective Investigation. *Diabetes Care* **2016**, *39*, 1371–1377, doi:10.2337/dc15-2373.
3. Waaijman, R.; Keukenkamp, R.; De Haart, M.; Polomski, W.P.; Nollet, F.; Bus, S.A. Adherence to Wearing Prescription Custom-Made Footwear in Patients With Diabetes at High Risk for Plantar Foot Ulceration. *Diabetes Care* **2013**, *36*, 1613–1618, doi:10.2337/dc12-1330.
4. Waaijman, R.; De Haart, M.; Arts, M.L.J.; Wever, D.; Verlouw, A.J.W.E.; Nollet, F.; Bus, S.A. Risk Factors for Plantar Foot Ulcer Recurrence in Neuropathic Diabetic Patients. *Diabetes Care* **2014**, *37*, 1697–1705, doi:10.2337/dc13-2470.
5. Bus, S.A.; Van Netten, J.J.; Kottink, A.I.; A Manning, E.; Spraul, M.; Woittiez, A.-J.; Van Baal, J.G. The efficacy of removable devices to offload and heal neuropathic plantar forefoot ulcers in people with diabetes: a single-blinded multicentre randomised controlled trial. *Int. Wound J.* **2017**, *15*, 65–74, doi:10.1111/iwj.12835.
6. Arts, M.; Haart, M.; Bus, S.; Bakker, J.; Hacking, H.; Nollet, F. Perceived usability and use of custom-made footwear in diabetic patients at high risk for foot ulceration. *J. Rehabil. Med.* **2014**, *46*, 357–362, doi:10.2340/16501977-1272.

2. Excluded Publications of Validation Set

2.1. Exclusion Reason: (Systematic) Review Without Meta-Analysis

7. Chatwin, K.E.; Abbott, C.A.; Boulton, A.J.; Bowling, F.L.; Reeves, N.D. The role of foot pressure measurement in the prediction and prevention of diabetic foot ulceration—A comprehensive review. *Diabetes/Metabolism Res. Rev.* **2019**, *36*, 1–14,, doi:10.1002/dmrr.3258.
8. Lazzarini, P.A.; Crews, R.T.; Van Netten, J.J.; Bus, S.A.; Fernando, M.E.; Chadwick, P.J.; Najafi, B. Measuring Plantar Tissue Stress in People With Diabetic Peripheral Neuropathy: A Critical Concept in Diabetic Foot Management. *J. Diabetes Sci. Technol.* **2019**, *13*, 869–880, doi:10.1177/1932296819849092.

2.2. Exclusion Reason: No Clinical Outcome

1. Maluf, K.; Mueller, M.J. Comparison of physical activity and cumulative plantar tissue stress among subjects with and without diabetes mellitus and a history of recurrent plantar ulcers. *Clin. Biomech.* **2003**, *18*, 567–575, doi:10.1016/s0268-0033(03)00118-9.
2. Frykberg, R.G.; A Lavery, L.; Pham, H.; Harvey, C.; Harkless, L.; Veves, A. Role of Neuropathy and High Foot Pressures in Diabetic Foot Ulceration. *Diabetes Care* **1998**, *21*, 1714–1719, doi:10.2337/diacare.21.10.1714.
3. Owings, T.M.; Apelqvist, J.; Stenström, A.; Becker, M.; Bus, S.A.; Kalpen, A.; Ulbrecht, J.S.; Cavanagh, P.R. Plantar pressures in diabetic patients with foot ulcers which have remained healed. *Diabet. Med.* **2009**, *26*,

1141–1146, doi:10.1111/j.1464-5491.2009.02835.x.
4. Arts, M.L.J.; Waaijman, R.; De Haart, M.; Keukenkamp, R.; Nollet, F.; Bus, S.A. Offloading effect of therapeutic footwear in patients with diabetic neuropathy at high risk for plantar foot ulceration. *Diabet. Med.* **2012**, *29*, 1534–1541, doi:10.1111/j.1464-5491.2012.03770.x.
5. Waaijman, R.; Keukenkamp, R.; De Haart, M.; Polomski, W.P.; Nollet, F.; Bus, S.A. Adherence to Wearing Prescription Custom-Made Footwear in Patients With Diabetes at High Risk for Plantar Foot Ulceration. *Diabetes Care* **2013**, *36*, 1613–1618, doi:10.2337/dc12-1330.
6. Arts, M.; Haart, M.; Bus, S.; Bakker, J.; Hacking, H.; Nollet, F. Perceived usability and use of custom-made footwear in diabetic patients at high risk for foot ulceration. *J. Rehabil. Med.* **2014**, *46*, 357–362, doi:10.2340/16501977-1272.

Supplementary file S2. Search Strings.

1. Search PubMed

The general search (1.1.) was combined with each of the foot-loading factor searches (1.2).

1.1. General Search

(((“Ulcer”[Mesh] OR ulcer*[tw]) AND (“Foot”[Mesh] OR foot[tw] OR feet[tw]) AND diabet*[tw]) OR (“Foot Ulcer”[Mesh] AND diabet*[tw]) OR diabetic foot[tw] OR diabetic feet[tw] OR ((“Foot Diseases”[Mesh] OR foot problem*[tw] OR foot disease*[tw]) AND diabet*[tw]))

1.2. Foot-Loading Factor Searches

1.2.1. Plantar Pressure

((pressure[mesh] AND foot[mesh]) OR (pressure[mesh] AND foot[tiab]) OR (pressure[mesh] AND feet[tiab]) OR (pressure[mesh] AND shoes[mesh]) OR plantar pressure [tw] OR barefoot pressure [tw] OR in-shoe pressure[tw] OR (plantar[tiab] AND stress*[tiab]) OR plantar stress*[tiab])

1.2.2. Weight-Bearing Activity

(step*[tw] OR step count[tw] OR stride*[tw] OR physical activit*[tiab] OR physical exercise*[tiab] OR daily activit*[tiab] OR (weightbearing[tiab] AND activit*[tiab]) OR (weight-bearing[tiab] AND activit*[tiab]))

1.2.3. Adherence

((Shoes[mesh] OR shoe*[tw] OR footwear[tw] OR cast*[tw] OR walker*[tw] OR (device*[tw] AND (removable[tw] OR non-removable[tw] OR offloading[tw] OR orthotic[tw]))) AND (patient compliance[mesh] OR complian*[tw] OR concordance[tw] OR adheren*[tw]))

2. Search EMBASE

The general search (2.1.) was combined with each of the foot-loading factor searches (2.2.).

2.1. General Search

1. Ulcer.ab,kw,ti
2. exp ulcer/
3. 1 or 2
4. exp foot/
5. (foot or feet).ab,kw,ti
6. 4 or 5
7. 3 and 6
8. diabet*.ab,kw,ti
9. 7 and 8
10. foot ulcer/ and diabet*.ab,kw,ti
11. diabetic foot/

12. ((diabet* and foot) or (diabet* and feet)).ab,kw,ti
13. 11 or 12
14. foot disease/
15. (foot disease* or foot problem*).ab,kw,ti
16. 14 or 15
17. diabet*.ab,kw,ti
18. 16 and 17
19. 9 or 10 or 13 or 18

2.2. Foot-Loading Factor Searches

2.2.1. Plantar Pressure

20. exp pressure/ and exp foot/
21. exp pressure/ and foot.ab,kw,ti
22. exp pressure/ and feet.ab,kw,ti
23. exp pressure/ and exp shoes/
24. plantar pressure.ab,kw,ti
25. barefoot pressure.ab,kw,ti
26. in-shoe pressure.ab,kw,ti
27. (plantar and stress*).ab,kw,ti
28. plantar stress*.ab,kw,ti
29. 20 or 21 or 22 or 23 or 24 or 25 or 26 or 27 or 28

2.2.2. Weight-Bearing Activity

30. steps.ab,kw,ti
31. step count.ab,kw,ti
32. stride*.ab,kw,ti
33. physical activit*.ab,kw,ti
34. physical exercise*.ab,kw,ti
35. daily activit*.ab,kw,ti
36. (weightbearing and activit*).ab,kw,ti
37. (weight-bearing and activit*).ab,kw,ti
38. 30 or 31 or 32 or 33 or 34 or 35 or 36 or 37

2.2.3. Adherence

39. exp shoes/
40. shoe*.ab,kw,ti
41. footwear.ab,kw,ti
42. cast*.ab,kw,ti
43. walker*.ab,kw,ti
44. device*.ab,kw,ti
45. removable.ab,kw,ti
46. non-removable.ab,kw,ti
47. offloading.ab,kw,ti
48. orthotic.ab,kw,ti
49. 45 or 46 or 47 or 48
50. 44 and 49
51. 39 or 40 or 41 or 42 or 43 or 50
52. exp patient compliance/
53. complian*.ab,kw,ti
54. concordance.ab,kw,ti
55. adheren*.ab,kw,ti
56. 52 or 53 or 54 or 55
57. 51 and 56
