# Supplementary material for: The Role of Foot-Loading Factors and Their Associations with Ulcer Development and Ulcer Healing in People with Diabetes: A Systematic Review
Source: J Clin Med. 2020 Nov 7;9(11):3591. doi: 10.3390/jcm9113591 (PMC7694972; doi:10.3390/jcm9113591)
Supplement: Supplementary file 1 [file jcm-09-03591-s001.zip › jcm-977657-supplementary table.pdf]

Table S1. Risk of bias assessment: QUIPS tool.

	Study Participation								Study Attrition						Prognostic Factor							Outcome Measurement				Study Confounding								Statistical Analysis and Reporting				
	Source or target population	Method identification population	Recruitment period	Place of recruitment	Inclusion and exclusion criteria	Adequate study population	Baseline characteristics	Summary study participation	Proportion baseline sample available	Attempts collecting information drop-outs	Reasons and impact drop-outs	Description drop-outs	No difference drop-outs and completers	Summary study attrition	Definition prognostic factor	Valid and reliable measurement	Continuous variables or cut-points	Method and setting	Proportion data available	Method missing data	Summary prognostic factor	Definition of outcome	Valid and reliable measurement	Method and setting	Summary outcome measurement	Important confounders measured	Definition confounders	Valid and reliable measurement	Method and setting	Method missing data	Confounders accounted for in study design	Confounders accounted for in analysis	Summary study confounding	Presentation of analytical strategy	Appropriate strategy model building	Statistical model adequate for study design	Reporting of results	Summary statistical analysis and reporting
Armstrong et al. 1998 [1]	+	-	-	-	±	?	+	H	+	+	+	+	+	L	+	+	+	+	+	+	L	-	?	+	H	+	+	+	+	+	+	+	L	±	+	+	+	L
Armstrong et al. 2004 [2]	+	±	-	-	±	?	+	H	+	+	+	+	+	L	-	+	+	+	+	+	M	-	-	?	H	±	±	+	+	+	±	-	H	+	-	-	+	M
Chantelau et al. 1994 [3]	±	-	-	-	+	?	±	H	+	+	+	+	+	L	+	±	+	+	+	+	M	-	±	+	H	±	-	?	+	+	-	-	H	+	-	-	+	M
Connor et al. 2004 [4]	+	-	+	-	+	±	+	M	?	?	?	?	?	H	±	-	-	?	+	+	H	±	±	±	H	+	±	±	+	+	-	-	H	+	-	-	+	M
Crews et al. 2016 [5]	+	-	+	±	+	?	+	M	+	-	+	+	±	L	+	+	+	+	±	±	L	±	±	+	M	±	+	+	+	-	+	+	M	+	±	+	±	M
Deschamps et al. 2016 [6]	+	-	+	+	+	?	+	L	+	+	+	+	+	L	+	+	+	+	+	+	L	±	±	+	M	+	+	±	+	+	-	-	H	+	-	-	+	M
Jarl et al. 2017 [7]	+	-	-	-	+	?	±	H	+	+	+	+	+	L	±	+	±	+	+	+	M	+	-	+	M	-	-	?	?	+	-	-	H	-	-	-	-	H
Kästenbauer et al. 2001 [8] and Grimm et al. 2004 [9]	+	-	-	-	+	+	+	M	+	-	-	-	-	H	+	+	+	+	+	-	L	+	-	+	M	+	+	+	+	±	±	±	M	+	±	±	+	M
Lavery et al. 2003 [10]	+	-	-	+	-	+	±	H	+	+	+	+	+	L	+	+	+	+	+	+	L	-	-	?	H	±	+	+	?	+	±	-	H	+	-	-	+	M
Ledoux et al. 2013 [11]	+	-	+	+	+	+	+	L	±	+	+	-	+	M	+	±	+	±	+	?	M	+	-	+	M	+	+	+	+	?	+	+	L	+	+	+	+	L
Lemaster et al. 2003 [12]	±	-	-	+	+	+	+	M	+	+	+	+	+	L	+	±	±	+	+	+	M	+	+	+	L	+	+	+	+	?	+	+	L	+	+	+	+	L
Mueller et al. 2013 [13]	+	+	+	+	+	+	+	L	+	+	+	+	+	L	+	±	+	+	+	+	L	+	+	+	L	+	+	+	+	-	-	-	H	+	-	-	+	M
Murray et al. 1996 [14]	+	-	-	+	+	?	±	M	+	+	+	+	+	L	±	+	-	+	+	+	M	±	±	+	M	±	±	+	+	+	-	-	H	+	-	-	?	M
Najafi et al. 2017 [15]	+	-	-	+	+	?	-	H	+	-	+	-	?	M	+	+	+	?	+	-	M	+	+	+	L	±	±	±	+	-	-	-	H	+	-	-	±	H
Pham et al. 2000 [16] and Caselli et al. 2002 [17]	+	-	-	+	-	+	+	M	+	+	+	+	+	L	+	+	+	+	+	+	L	-	?	?	H	+	+	+	+	+	+	+	L	+	+	+	+	L
Qiu et al. 2015 [18]	+	+	+	+	+	+	+	L	+	+	+	+	+	L	±	?	+	+	+	+	M	-	?	+	H	+	+	+	+	+	±	±	M	±	-	-	+	H
Schneider et al. 2019 [19]	+	-	-	+	+	-	+	H	+	-	-	-	±	M	+	+	+	+	+	?	L	-	+	?	H	+	+	+	+	±	-	-	H	+	-	-	+	M
Ulbrecht et al. 2014 [20]	+	+	-	+	+	+	+	L	+	+	+	+	+	L	+	+	+	+	+	+	L	+	+	+	L	+	±	+	+	?	-	-	M	+	-	-	+	M
Van Netten et al. 2018 [21]	+	-	+	+	+	±	+	L	+	+	+	+	+	L	+	+	+	+	+	+	L	+	±	+	M	+	±	±	±	+	-	-	M	+	±	±	+	M
Waaijman et al. 2014 [22]	+	-	-	-	+	+	+	L	+	?	-	+	+	M	+	+	+	+	?	-	M	+	+	+	L	+	+	+	+	+	+	+	L	+	+	+	+	L

+ = yes (green), ± = partial (orange), - = no (red), ? = unsure (yellow). Summary domain score: L = low (green), M = moderate (orange) and H = high risk of bias (red).

Table S2. Risk of bias assessment: 21-item score.

	1. Appropriate Definition	2. Appropriate Study Population	4. Description Intervention	5. Other Components of Care	8. Power Calculation	9. Primary Outcome of Direct Clinical Relevance	10. Assessor Primary Outcome Blinded to Group Allocation Primary Outcome	11. Clinical Researcher Blinded to Group	12. Completion of Recruitment	13.Primary Outcome in >75% of those Recruited	15. Appropriate Statistical Methods are Used	16. Cohort Performance as Expected in Clinical Practice	17. Comparable Results from Participating Centers	18. Free from Errors of Reporting	19. Balanced Discussion	20. Conclusions Supported by Findings	21. No Commercial Interests	Total Score* (Out of 17)
Armstrong et al. 1998 [1]	-	+	+	+	-	+	-	-	?	+	+	+	+	+	-	+	+	11
Armstrong et al. 2004 [2]	-	+	+	+	-	+	?	?	?	+	+	?	?	?	-	+	+	8
Chantelau et al. 1994 [3]	-	+	+	+	-	+	?	?	?	+	+	+	+	+	-	+	+	11
Connor et al. 2004 [4]	-	+	?	-	-	?	?	?	?	+	+	+	+	+	+	+	+	9
Crews et al. 2016 [5]	+	+	+	+	-	-	-	-	?	+	+	?	-	+	+	+	+	10
Deschamps et al. 2016 [6]	-	+	+	-	-	+	?	?	?	+	+	+	?	+	-	+	+	9
Jarl et al. 2017 [7]	+	+	+	+	-	+	?	?	?	+	-	+	+	+	+	+	+	12
Kästenbauer et al. 2001 [8] and Grimm et al. 2004 [9]	+	+	+	+	-	+	?	?	?	+	+	+	+	+	+	+	+	13
Lavery et al. 2003 [10]	-	+	+	+	-	+	?	?	?	+	+	?	?	+	+	+	+	10
Ledoux et al. 2013 [11]	+	+	+	?	-	+	?	?	?	+	+	?	+	+	+	+	+	11
Lemaster et al. 2003 [12]	+	+	+	-	-	+	+	+	?	+	+	?	?	+	?	+	+	11
Mueller et al. 2013 [13]	+	+	+	-	+	+	+	+	+	+	+	?	+	+	+	+	+	15
Murray et al. 1996 [14]	-	+	+	-	-	+	-	-	?	+	+	+	+	+	-	+	+	10
Najafi et al. 2017 [15]	+	+	+	+	-	+	+	?	?	+	+	?	-	+	+	+	+	12
Pham et al. 2000 [16] and Caselli et al. 2002 [17]	-	+	+	-	-	+	?	?	?	+	+	+	?	+	-	+	+	9
Qiu et al. 2015 [18]	-	+	+	-	-	+	?	?	?	+	+	-	+	+	-	?	+	8
Schneider et al. 2019 [19]	-	+	+	-	-	+	?	?	?	+	+	+	+	+	+	+	+	11
Ulbrecht et al. 2014 [20]	+	+	+	+	+	+	+	+	+	+	+	+	+	+	+	+	-	16
Van Netten et al. 2018 [21]	+	+	+	+	-	+	+	-	?	+	+	+	?	+	+	+	+	13
Waaijman et al. 2014 [22]	+	+	+	-	-	+	+	-	?	+	+	+	?	+	+	+	+	12

Question 3, 6, 7 and 14 of the 21-item score are excluded (see Methods), so 17 items in total. + = yes (green), ? = unsure/unknown (orange), - = no (red). * Total score is sum of +'s out of 17.

Table S3. Risk of bias assessment: SIGN.

	Internal Validity												Overall Assessment	
	1.1 Clear research question and listed inclusion / exclusion criteria	1.2 Comprehensive literature search	1.3 Two people selected studies	1.4 Two people extracted data	1.5 Status of publication was not used as an inclusion criterion	1.6 Excluded studies are listed	1.7 Relevant characteristics of included studies are provided	1.8 Assessment and reporting of quality of included studies	1.9 Scientific quality of included studies appropriately used	1.10 Appropriate methods used to combine individual study findings	1.11 Likelihood of publication bias was assessed appropriately	1.12 Conflicts of interest are declared	2.1 Overall assessment of the methodological quality	2.2 Results are directly applicable to the patient group targeted
Crawford et al. 2007 [23]	yes	yes	yes	can't say	yes	yes	no	yes	yes	yes	no	no	high quality	yes

Table S4. Plantar pressure - ulcer development.

Reference	Population	Risk of Bias	Ulcer Outcome	Factor + Outcomes	Conclusion
Crawford et al. 2007 [23]<br>Meta-analysis	Pham et al. 2000 and Lavery et al. 2003	SIGN: High quality study	Pham et al. 2000<br>Ulcerated: 29% (n = 73)<br>Lavery et al. 2003<br>Ulcerated: 16% (n = 263)<br>Total<br>Ulcerated: 21% (n = 336)	Barefoot PP:<br>Pham et al. 2000<br>Weight: 36.59%<br>SMD: 0.62, 95% CI: (0.35, 0.90)<br>Lavery et al. 2003<br>Weight: 63.41%<br>SMD: 0.38, 95% CI: (0.25, 0.52)<br>Total<br>Weight: 100%<br>SMD: 0.47, 95% CI: (0.24; 0.77)	Associated (Z = 4.05, p < 0.0001)
Pham et al. 2000 [16] and Caselli et al. 2002 [17] *<br>Prospective cohort study<br>Period: not presented<br>Location: Boston, MA; San Antonio, TX; San Francisco, CA, USA (multicenter)	N: 248<br>Male: 50%<br>Age: 58 ± 12 years<br>Type 2: 80%<br>Neuropathy: 84%<br>Risk classification: not presented	QUIPS: M-L-L-H-L-L<br>21-item score: 9/17	At 30 (6–40) months:<br>Ulcerated: 29% (n = 73)	Barefoot PP (kPa):<br>Ulcerated: 706 ± 373<br>Non-ulcerated: 522 ± 255	Associated (p < 0.001)
			At 30 (6–40) months:<br>≥588 kPa: 45% ulcerated (n = 173)<br><588 kPa: 34% ulcerated (n = 75)	Barefoot cut-off PP ≥588 kPa	Associated (OR: 3.2, 95% CI: (2.0; 5.1), p < 0.001 (univariate), OR: 2.0, 95% CI: (1.2; 3.3), p = 0.007 (multivariate Pham et al. 2000) and OR: 1.8, 95% CI: (1.0-3.0), p = 0.03 (multivariate Caselli et al. 2002))
			At 30 (6–40) months:<br>Number of participants and percentage ulceration per group not presented	Barefoot forefoot-to-rearfoot PP ratio >2	Associated (OR: 2.7, 95% CI: (1.7; 4.3), p = 0.001 (univariate) and OR: 1.8, 95% CI: (1.1; 3.2), p = 0.03 (multivariate))
Lavery et al. 2003 [10]<br>Prospective cohort study<br>Period: not presented<br>Location: San Antonio, TX, USA (monocenter)	N: 1666<br>Male: 50.4%<br>Age: 69.1 ± 11.1 years<br>Type 2: not presented<br>Neuropathy: not presented<br>Risk classification: not presented	QUIPS: H-L-L-H-H-M<br>21-item score: 10/17	At 24 (20–29) months:<br>Ulcerated: 16% (n = 263)	Barefoot PP (kPa):<br>Ulcerated: 955 ± 264<br>Non-ulcerated: 851 ± 273	Associated (p < 0.001)
			At 24 (20–29) months:<br>>875 kPa: 17% ulcerated<br><875 kPa: 10% ulcerated<br>Number of participants per group not presented †	Cut-off PP: >875 kPa	Associated (OR: 2.0, 95% CI: (1.4; 2.9), p = 0.0001)
Kästenbauer et al. 2001 [8] and Grimm et al. 2004 [9] ‡<br>Prospective cohort study<br>Period: 1994–1998<br>Location: Vienna, Austria (monocenter)	N: 187<br>Male: 55%<br>Age: 58.6 years<br>Type 2: 100%<br>Neuropathy: 38%<br>Risk classification: not presented	QUIPS: M-H-L-M-M-M<br>21-item score: 13/17	At 3.6 years (mean):<br>Ulcerated: 5% (n = 10)	Barefoot PP (kPa):<br>Ulcerated: 785 (705; 1155)<br>Non-ulcerated: 605 (430; 800)	Associated (p < 0.01)
			At 3.6 years (mean):<br>Number of participants and percentage ulceration per group (>2 SD / <2 SD) not presented	Barefoot cut-off PP >2 SD compared to a healthy participant in at least one forefoot region	Associated (RR: 6.3, 95% CI: (1.2; 32.7), p = 0.0291)
Ulbrecht et al. 2014 [20]<br>Prospective randomized controlled trial<br>Period: not presented<br>Location: PA; IL; CA; AZ; OH; TX; CO, USA (multicenter)	N: 130<br>Male: 78%<br>Age: 59.5 years<br>Type 2: not presented<br>Neuropathy: 100%<br>Risk classification: IWGDF 3: 100%	QUIPS: L-L-L-L-M-M<br>21-item score: 16/17	At 15 months:<br>Ulcerated: 17% (n = 22)<br>Non-ulcerative lesions: 25% (n = 32)	Barefoot PP (kPa):<br>Ulcerated: 1131<br>Non-ulcerative lesions: 1042<br>Non-ulcerated: 984	Associated (p = 0.04)

Deschamps et al. 2016 [6]<br>Retrospective cohort study<br>Period: 2010–2011<br>Location: Leuven; Aalst, Belgium (multicenter)	*N*: 97, 194 feet<br>*Numbers below for feet:*<br>Male: 68%<br>Age: 61.6 years<br>Type 2: 72%<br>Neuropathy: 51%<br>Risk classification: IWGDF 0: 41%, IWGDF 1: 3%, IWGDF 2: 12%, IWGDF 3: 44%	QUIPS: L-L-L-M-H-M<br>21-item score: 9/17	At 42.7 months (mean):<br>Ulcerated: 7% ($n = 7$)	Barefoot PP (kPa):<br>Ulcerated: 1332.4 ± 207.8<br>Non-ulcerated: 814.8 ± 247.6	Associated ($r = 0.462$, $p < 0.001$) [§]
Murray et al. 1996 [14]<br>Prospective cohort study<br>Period: not presented<br>Location: Manchester, UK (multicenter)	*N*: 63<br>Male: 68%<br>Age: 62 (52; 67) years<br>Type 2: 60%<br>Neuropathy: 100%<br>Risk classification: not presented	QUIPS: M-L-M-M-H-M<br>21-item score: 10/17	At 15.4 (10–22) months:<br>Ulcerated: 10% ($n = 6$)	Barefoot PP (kPa):<br>Ulcerated: 1451 (1098; 1952)<br>Non-ulcerated: 1677 (1461; 2099)	Not associated ($p = 0.14$)
			At 15.4 (10–22) months:<br>Incidence of ulcers by individual area of the foot:<br><981 kPa: 0.3% ulcerations ($n = 3/886$)<br>≥981 kPa: 1.6% ulcerations ($n = 4/248$)	Cut-off PP: ≥981 kPa	Associated (RR: 4.7, 95% CI: (1.2; 18.9), $p = 0.04$)
Qiu et al. 2015 [18]<br>Prospective before and after study<br>Period: 2012<br>Location: Shijazhuang, Hebei, China (monocenter)	*N*: 65<br>Male: 45%<br>Age: 59 (40–78) years<br>Type 2: 100%<br>Neuropathy: not presented<br>Risk classification: not presented	QUIPS: L-L-M-H-M-H<br>21-item score: 8/17	At 2 years:<br>Ulcerated: 3% ($n = 2$)	Barefoot PP change over 2 years in the metatarsals:<br>Barefoot PP changes per group not presented.	Not associated ($p > 0.05$)
				Barefoot PTI change over 2 years in the metatarsals:<br>Barefoot PTI changes per group not presented.	Not associated ($p > 0.05$)
Waaijman et al. 2014 [22] [‖]<br>Prospective randomized controlled trial<br>Period: 2007–2010<br>Location: the Netherlands (multicenter)<br>Shoes: custom-made orthopedic footwear with or without pressure optimization	*N*: 171<br>Male: 82.5%<br>Age: 63.3 ± 10.1 years<br>Type 2: 71.3%<br>Neuropathy: 100%<br>Risk classification: IWGDF 3: 100%	QUIPS: L-M-M-L-L-L<br>21-item score: 12/17	At 18 months:<br>Plantar ulcers: 42% ($n = 71$)	Barefoot PP (kPa):<br>Plantar ulcers: 1042 ± 260<br>No plantar ulcers: 935 ± 307	Associated (OR: 1.18, 95% CI: (1.09; 1.27), $p < 0.0001$)
				In-shoe PP (measured quarterly during follow-up) (kPa):<br>Plantar ulcers: 261 ± 83<br>No plantar ulcers: 246 ± 77	Not associated (OR: 1.21, 95% CI: (0.95; 1.53), $p = 0.120$)
			At 18 months:<br>Ulcers from unrecognized trauma: 24% ($n = 41$)	Barefoot PP (kPa):<br>Ulcers from unrecognized trauma: 849 ± 375<br>No ulcers from unrecognized trauma: 699 ± 393	Associated (OR: 1.12, 95% CI: (1.05; 1.21), $p = 0.001$ (univariate), OR: 1.11, 95% CI: (1.00; 1.22), $p = 0.040$ (multivariate))
				In-shoe PP (measured quarterly during follow-up) (kPa)<br>Ulcers from unrecognized trauma: 212 ± 99<br>No ulcers from unrecognized trauma: 178 ± 82	Associated (OR: 1.38, 95% CI: (1.05; 1.81), $p = 0.023$)
Ledoux et al. 2013 [11]<br>Prospective cohort study<br>Period: 1996–2002	*N*: 591<br>Male: 98.1%<br>Age: 67 years	QUIPS: L-M-M-M-L-L<br>21-item score: 11/17	At 2.4 (0–6.2) years:<br>Ulcerated: 8% ($n = 47$)	In-shoe PP (kPa):<br>Ulcerated: 219<br>Non-ulcerated: 194	Not associated (HR: 1.27, 95% CI: (0.89; 1.74), $p = 0.171$)

Location: WA, USA (monocenter)<br>Shoes: "usual" shoes	Type 2: not presented<br>Neuropathy: 50%<br>Risk classification: not presented			In-shoe PTI (kPa·s):<br>Ulcerated: 89<br>Non-ulcerated: 79	Not associated (HR: 1.25, 95% CI: (0.94; 1.52), $p = 0.115$)

All plantar pressures are measured during baseline unless otherwise mentioned. Results expressed as mean ± standard deviation, median (quartile 1; quartile 3) or mean (minimum-maximum). Abbreviations: N = number of participants, type 2 = type 2 diabetes, PP = peak pressure, PTI = peak pressure gradient, kPa = kilo Pascal, kPa·s = kilo Pascal times seconds, 95% CI = 95% confidence interval, OR = odds ratio, RR = relative ratio, HR = hazard ratio, SMD = standardized mean difference, r = Pearson correlation coefficient. Z = standard score of overall effect. Quips domains: (1) Study participation, (2) Study attrition, (3) Prognostic factor measurement, (4) Outcome measurement, (5) Study confounding, (6) Statistical analysis and reporting. L = low, M = moderate and H = high risk of bias. * 10 participants less in Caselli et al. 2002 than in Pham et al. 2000, reason of exclusion undefined in Caselli et al. 2002. The results of "Barefoot PP (kPa)" can be found in Pham et al. 2000, the results of "Barefoot cut-off PP ≥588 kPa" can be found in both Pham et al. 2000 and Caselli et al. 2002 and the results of "Barefoot forefoot-to-rearfoot PP ratio >2" can be found in Caselli et al. 2002. † Lavery et al. 2003 eliminated participants without a loss of protective sensation for this association, but the number of participants for this group is not presented. ‡ 36 participants less in Grimm et al. 2004 than in Kästenbauer et al. 2001, 151 of the 187 participants completed the study per protocol and that constituted the basis of the analysis of Grimm et al. 2004. The result of "Barefoot cut-off PP > 2 SD" can be found in Kästenbauer et al. 2001 and the results of "Barefoot PP (kPa)" can be found in Grimm et al. 2004. § Deschamps et al. 2016, analysis based on 109 participants (218 feet) and 7 ulcerations using data provided by the authors. ‖ Waaijman et al. 2014, plantar ulcers = any plantar ulcer; ulcers from unrecognized trauma = ulcer recurrence at previous ulcer location and not to be the result of acute trauma. A significance level of $p < 0.10$ was used.

Table S5. Weight-bearing activity - ulcer development.

Reference	Population	Risk of Bias	Ulcer Outcome	Factor + Outcomes	Conclusion
Armstrong et al. 2004 [2]<br>Prospective cohort study<br>Period: not presented<br>Location: USA (not presented)	*N*: 100<br>Male: 95%<br>Age: 68.5 ± 10.0 years<br>Type 2: not presented<br>Neuropathy: 100%<br>Risk classification: IWGDF 2: 68% and IWGDF 3: 32%	QUIPS: H-L-M-H-H-M<br>21-item score: 8/17	At 37.1 ± 12.3 weeks (mean):<br>Ulcerated: 8% ($n = 8$)	Average daily activity* (entire study period):<br>Ulcerated: 809.0 ± 612.2<br>Non-ulcerated: 1394.5 ± 868.5	Associated ($p = 0.03$)
				Coefficient of variation in daily activity (%)<br>Ulcerated: 96.4 ± 50.3<br>Non-ulcerated: 44.7 ± 15.4	Associated ($p = 0.0001$)
Lemaster et al. 2003 [12]<br>Prospective cohort study<br>Period: 1997–1998<br>Location: WA, USA (multicenter)	*N*: 390<br>Male: 77%<br>Age: 62.5 ± 9.0 years<br>Type 2: not presented<br>Neuropathy: 58%<br>Risk classification: IWGDF 3: 100%	QUIPS: M-L-M-L-L-L<br>21-item score: 11/17	At 2 years:<br>Least active: 17% ulcerated ($n = 22/133$)<br>Moderately active: 13% ulcerated ($n = 18/134$)<br>Most active: 13% ulcerated ($n = 16/123$)	Activity group (activity measured every 17th week during follow-up):<br>Least active: <4.5 h per day<br>Moderately active: 4.5–7.4 h per day<br>Most active: ≥7.5 h per day	Not associated ($p > 0.05$)
Waaijman et al. 2014 [22] †<br>Prospective randomized controlled trial<br>Period: 2007–2010<br>Location: the Netherlands (multicenter)	*N*: 171<br>Male: 82.5%<br>Age: 63.3 ± 10.1 years<br>Type 2: 71.3%<br>Neuropathy: 100%<br>Risk classification: IWGDF 3: 100%	QUIPS: L-M-M-L-L-L<br>21-item score: 12/17	At 18 months:<br>Plantar ulcers: 42% ($n = 71$)	Steps per day (7-day period <3 months after baseline):<br>Plantar ulcers: 6476 ± 2574<br>No plantar ulcers: 6874 ± 3980	Not associated (OR: 0.99, 95% CI: (0.97; 1.01), $p = 0.360$)
				Variation in steps per day (7-day period <3 months after baseline):<br>Plantar ulcers: 2136 ± 1098<br>No plantar ulcers: 2552 ± 1586	Associated (OR: 0.95, 95% CI: (0.90; 0.99), $p = 0.023$ (univariate), OR: 0.93, 95% CI: (0.89; 0.99), $p = 0.012$ (multivariate))

			At 18 months:<br>Ulcers from unrecognized trauma: 24% (n = 41)	Steps per day (7-day period <3 months after baseline):<br>Ulcers from unrecognized trauma: 6418 ± 2662<br>No ulcers from unrecognized trauma: 6808 ± 3714	Not associated (OR: 0.99, 95% CI: (0.97; 1.01), p = 0.404)
				Variation in steps per day (7-day period <3 months after baseline):<br>Ulcers from unrecognized trauma: 2124 ± 1156<br>No ulcers from unrecognized trauma: 2468 ± 1494	Associated (OR: 0.96, 95% CI: (0.91; 1.01), p = 0.096 (univariate), OR: 0.91, 95% CI: (0.86; 0.96), p = 0.001 (multivariate))
Mueller et al. 2013 [13]<br>Prospective randomized controlled trial<br>Period: 2009–2011<br>Location: WA, USA (monocenter)	*N*: 29<br>Male: 59%<br>Age: 64.5 ± 12.5 years<br>Type 2: 100%<br>Neuropathy: 100%<br>Risk classification: not presented	QUIPS: L-L-L-L-H-M<br>21-item score: 15/17	At 12 weeks:<br>Ulcerated: 10% (n = 3)	Steps per day (baseline)<br>Ulcerated: 5360 ± 1673<br>Non-ulcerated: 5717 ± 1751	Number of ulcerations too low to assess association.
				Steps per day (12 weeks)<br>Ulcerated: 4654 ± 1211<br>Non-ulcerated: 5941 ± 1751	
Schneider et al. 2019 [19]<br>Prospective mixed methods study<br>Period: not presented<br>Location: IL, USA (monocenter)	*N*: 12<br>Male: 33.3%<br>Age: 59.92 ± 8.68 years<br>Type 2: 91.7%<br>Neuropathy: 100%<br>Risk classification: not presented	QUIPS: H-M-L-H-H-M<br>21-item score: 11/17	At 20 weeks:<br>Ulcerated: 8% (n = 1)	Steps per day (baseline):<br>Ulcerated: 1883.00<br>Non-ulcerated: 3871.80 ± 1367.88	Number of ulcerations too low to assess association.
				Steps per day (after 20 weeks):<br>Ulcerated: 2957.00<br>Non-ulcerated: 4342.00 ± 1292.09	

Results expressed as mean ± standard deviation. Abbreviations: N = number of participants, type 2 = type 2 diabetes, CI = 95% confidence interval, OR = odds ratio. Quips domains: (1) Study participation, (2) Study attrition, (3) Prognostic factor measurement, (4) Outcome measurement, (5) Study confounding, (6) Statistical analysis and reporting. L = low, M = moderate and H = high risk of bias. * Armstrong et al. 2004, daily activity measured in activity units, which is an undefined measure in Armstrong et al. 2004. † Waaijman et al. 2014, plantar ulcers = any plantar ulcer; ulcers from unrecognized trauma = ulcer recurrence at previous ulcer location and not to be the result of acute trauma. A significance level of $p < 0.10$ was used.

Table S6. Footwear adherence - ulcer development.

Reference	Population	Risk of Bias	Ulcer Outcome	Factor + Outcomes	Conclusion
Chantelau et al. 1994 [3]<br>Prospective cohort study<br>Period: not presented<br>Location: Düsseldorf, Germany (monocenter)	*N*: 51<br>Male: 59%<br>Age: 63 (60; 67) years<br>Type 2: 71%<br>Neuropathy: not presented<br>Risk classification: IWGDF 3: 100%	QUIPS: H-L-M-H-H-M<br>21-item score: 11/17	At 4 years:<br>≥9.6 / 16 h per day: 54% ulcerated (n = 20/37)<br><9.6 / 16 h per day: 100% ulcerated (n = 14/14)	Cut-off adherence ≥9.6/16 h per day	Associated (p = 0.0002)
Connor et al. 2004 [4]<br>Retrospective cohort study<br>Period: 1996-not presented<br>Location: Hereford, UK (monocenter)	*N*: 83<br>Male: 67%<br>Age: 57.0 years<br>Type 2: 69%<br>Neuropathy: 100%<br>Risk classification: IWGDF 3: 100%	QUIPS: M-H-H-H-H-M<br>21-item score: 9/17	After minimum 2 years:<br>≥3.5 ulcers per foot per 10 years: 64% of ulcers (n = 207), 37% of participants (n = 31)<br><3.5 ulcers per foot per 10 years: 36% of ulcers (n = 114), 63% of participants (n = 52)	% of participants non-adherent to footwear:<br>≥3.5 ulcers per foot per 10 years: 55<br><3.5 ulcers per foot per 10 years: 35	Associated (p = 0.037)
Waaijman et al. 2014 [22] *<br>Prospective randomized controlled trial<br>Period: 2007–2010<br>Location: the Netherlands (multicenter)	*N*: 171<br>Male: 82.5%<br>Age: 63.3 ± 10.1 years<br>Type 2: 71.3%<br>Neuropathy: 100%<br>Risk classification: IWGDF 3: 100%	QUIPS: L-M-M-L-L-L<br>21-item score: 12/17	At 18 months:<br>Plantar ulcers: 42% (n = 71)	% adherence (7-day period < 3 months after baseline):<br>Plantar ulcers: 73.1 ± 24.7<br>No plantar ulcers: 72.7 ± 24.1	Not associated (OR: 1.00, 95% CI: (0.99; 1.01), p = 0.989)
			At 18 months:<br>Ulcers from unrecognized trauma: 24% (n = 41)	% adherence:<br>Ulcers from unrecognized trauma: 72.2 ± 26.2<br>No ulcers from unrecognized trauma: 73.1 ± 23.7	Not associated (OR: 1.00, 95% CI: (0.98; 1.01), p = 0.823)

Results expressed as mean ± standard deviation or median (quartile 1; quartile 3). Abbreviations: N = number of participants, type 2 = type 2 diabetes, CI = 95% confidence interval, OR = odds ratio. Quips domains: (1) Study participation, (2) Study attrition, (3) Prognostic factor measurement, (4) Outcome measurement, (5) Study confounding, (6) Statistical analysis and reporting. L = low, M = moderate and H = high risk of bias. * Waaijman et al. 2014, plantar ulcers = any plantar ulcer; ulcers from unrecognized trauma = ulcer recurrence at previous ulcer location and not to be the result of acute trauma. A significance level of $p < 0.10$ was used.

Table S7. Cumulative plantar tissue stress - ulcer development.

Reference	Population	Risk of Bias	Ulcer Outcome	Factor + Outcomes	Conclusion
Waaijman et al. 2014 [22] *<br>Prospective randomized controlled trial<br>Period: 2007–2010<br>Location: the Netherlands (multicenter)<br>Shoes: custom-made orthopedic footwear with or without pressure optimization	*N*: 171<br>Male: 82.5%<br>Age: 63.3 ± 10.1 years<br>Type 2: 71.3%<br>Neuropathy: 100%<br>Risk classification: IWGDF 3: 100%	QUIPS: L-M-M-L-L-L<br>21-item score: 12/17	At 18 months:<br>Plantar ulcers: 42% (n = 71)	Cumulative plantar tissue stress (7-day period <3 months after baseline) (MPa·s/day):<br>Plantar ulcers: 715 ± 538<br>No plantar ulcers: 652 ± 436	Not associated (OR: 1.00, 95% CI: (1.00; 1.00), p = 0.453)
				% of participants with in-shoe PP at plantar foot <200 kPa and adherence >80%:<br>Plantar ulcers: 9.0<br>No plantar ulcers: 10.2	Associated (OR: 0.47, 95% CI: (0.26; 0.85), p = 0.012)
			At 18 months:<br>Ulcers from unrecognized trauma: 24% (n = 41)	Cumulative plantar tissue stress (MPa·s/day):<br>Ulcers from unrecognized trauma: 423 ± 292<br>No ulcers from unrecognized trauma: 361 ± 279	Not associated (OR: 1.00, 95% CI: (1.00; 1.00), p = 0.162)
				% of participants with in-shoe PP at previous ulcer <200 kPa and adherence >80%:<br>Ulcers from unrecognized trauma: 17.9	Associated (OR: 0.5, 95% CI: (0.28; 0.89), p = 0.019 (univariate), (OR:

No ulcers from unrecognized trauma: 27.3	0.43, 95% CI: (0.20; 0.94), $p = 0.033$ (multivariate))

Results expressed as mean ± standard deviation. Abbreviations: N = number of participants, type 2 = type 2 diabetes, MPa·s/day = MegaPascal times seconds per day, CI = 95% confidence interval, OR = odds ratio. Quips domains: (1) Study participation, (2) Study attrition, (3) Prognostic factor measurement, (4) Outcome measurement, (5) Study confounding, (6) Statistical analysis and reporting. L = low, M = moderate and H = high risk of bias. * Waaijman et al. 2014, plantar ulcers = any plantar ulcer; ulcers from unrecognized trauma = ulcer recurrence at previous ulcer location and not to be the result of acute trauma. A significance level of $p < 0.10$ was used.

Table S8. Plantar pressure - ulcer healing.

Reference	Population	Risk of bias	Ulcer outcome	Factor + outcomes	Conclusion
Armstrong et al. 1998 [1]<br>Prospective cohort study<br>Period: not presented<br>Location: San Antonio, TX, USA (monocenter)	*N*: 25<br>Male: 68%<br>Age: 52.4 ± 11.6 years<br>Type 2: 84%<br>Neuropathy: 100%<br>Ulcer classification: Meggitt-Wagner grade 1 in depth: 100%<br>Location: hallux: 4%, MTP1: 52%, MTP2: 12%, MTP3: 8%, MTP5: 12%, lateral midfoot: 4% and heel: 8%<br>Area: 7.7 ± 4.0 cm²	QUIPS: H-L-L-H-L-L<br>21-item score: 11/17	Days until ulcer healing:<br>>990 kPa: 53.4 ± 31.4<br><990 kPa: 33.1 ± 13.0<br>Number of participants per group not presented	Barefoot cut-off PP > 990 kPa	Not associated ($p = 0.05$)
			Mean days until ulcer healing:<br>38.8 ± 21.3	Mean barefoot PP (kPa):<br>927 ± 143	Associated (95% CI: (0.1; 0.91), $p < 0.03$)
Van Netten et al. 2018 [21]<br>Prospective cohort study<br>Period: 2004–2013<br>Location: the Netherlands and Germany (multicenter)<br>Device: removable knee-high or ankle-high offloading device	*N*: 31<br>Male: 81%<br>Age: 60 ± 12.6 years<br>Type 2: 94%<br>Neuropathy: 100%<br>Ulcer classification: 68% Texas 1A and 32% Texas 2A<br>Location: hallux: 45%, MTP1: 26%, MTP2–5: 29%<br>Area: 1.1 ± 0.9 cm²	QUIPS: L-L-L-M-M-M<br>21-item score: 13/17	At 12 weeks:<br>Healed: 68% ($n = 21$)	In-device PP (2 weeks after baseline) (kPa)<br>Healed: 108 ± 56<br>Non-healed: 107 ± 57	Not associated (95% CI: (-45; 44), $p = 0.97$, $d = 0.02$)
				In-device PTI (2 weeks after baseline) (kPa·s)<br>Healed: 45 ± 29<br>Non-healed: 38 ± 17	Not associated (95% CI: (-28; 13), $p = 0.44$, $d = 0.29$)
Jarl et al. 2017 [7]<br>Prospective cohort study<br>Period: not presented<br>Location: Uppsala, Sweden (monocenter)<br>Device: non-removable ankle-high device	*N*: 7<br>Male: 100%<br>Age: 63 (35–80) years<br>Type 2: 100%<br>Neuropathy: 100%<br>Ulcer classification: Wagner grade 1: 57%, Wagner grade 2: 43%<br>Location: MTP1: 57%, MTP3: 14%, MTP4: 14%, MTP5: 14%<br>Area: 0.5 (0.2–2.0) cm²	QUIPS: H-L-M-M-H-H<br>21-item score: 12/17	Days until ulcer healing:<br>49 (8–160)	In-device PP (kPa):<br>120 (62–192)	Not associated ($\varrho = -0.600$, $p = 0.208$) *

All plantar pressures are measured during baseline unless otherwise mentioned. Results expressed as mean ± standard deviation or median (minimum–maximum). Abbreviations: N = number of participants, type 2 = type 2 diabetes, PP = peak pressure, PTI = peak pressure gradient, kPa = kilo Pascal, kPa·s = kilo Pascal times seconds, ϱ = spearman's rho, 95% CI = confidence interval, d = effect size. Quips domains: (1) Study participation, (2) Study attrition, (3) Prognostic factor measurement, (4) Outcome measurement, (5) Study confounding, (6) Statistical analysis and reporting. L = low, M = moderate and H = high risk of bias. * Jarl et al. 2017, analysis based on 6 participants using anonymized data provided by the author.

Table S9. Weight-bearing activity - ulcer healing.

Reference	Population	Risk of bias	Ulcer outcome	Factor + outcomes	Conclusion
Najafi et al. 2017 [15]<br>Prospective randomized controlled trial<br>Period: not presented<br>Location: Doha, Qatar and AZ, USA (multicenter)	*N*: 49<br>Male: 92%<br>Age: 53.7 ± 7.7 years<br>Type 2: not presented<br>Neuropathy: 100%<br>Ulcer classification: noninfected, nonischemic, plantar neuropathic ulcers<br>Location: not presented<br>Area: 8.14 (0.16–39.0) cm²	QUIPS: H-M-M-L-H-H<br>21-item score: 12/17	At 12 weeks:<br>Healed: 51% (*n* = 22) *	Steps per day (baseline):<br>Healed: 5304 ± 3567<br>Non-healed: 4312 ± 2658	Not associated (*p* = 0.398)
				Steps per day (last visit or week before healing):<br>Healed: 2595 ± 2056<br>Non-healed: 5586 ± 3186	Not associated (*p* = 0.104)
				Standing % of 24 hours (baseline):<br>Healed: 9.8 ± 5.3<br>Non-healed: 11.2 ± 6.7	Not associated (*p* = 0.518)
				Standing % of 24 hours (last visit or week before healing):<br>Healed: 5.7 ± 4.0<br>Non-healed: 11.4 ± 3.9	Associated (OR: 0.663, *p* = 0.013)
Van Netten et al. 2018 [21]<br>Prospective cohort study<br>Period: 2004–2013<br>Location: the Netherlands and Germany (multicenter)	*N*: 31<br>Male: 81%<br>Age: 60 ± 12.6 years<br>Type 2: 94%<br>Neuropathy: 100%<br>Ulcer classification: 68% Texas 1A and 32% Texas 2A<br>Location: hallux: 45%, MTP1: 26%, MTP2–5: 29%<br>Area: 1.1 ± 0.9 cm²	QUIPS: L-L-L-M-M-M<br>21-item score: 13/17	At 12 weeks:<br>Healed: 68% (*n* = 21)	Steps per day (2 weeks after baseline):<br>Healed: 7222 ± 3272<br>Non-healed: 9706 ± 6520	Not associated (95% CI: (-541; 3023), *p* = 0.26, *d* = 0.48)

Results expressed as mean ± standard deviation. Abbreviations: N = number of participants, type 2 = type 2 diabetes, CI = 95% confidence interval, OR = odds ratio, d = effect size. Quips domains: (1) Study participation, (2) Study attrition, (3) Prognostic factor measurement, (4) Outcome measurement, (5) Study confounding, (6) Statistical analysis and reporting. L = low, M = moderate and H = high risk of bias. * Najafi et al. 2017, 6 participants were excluded due to development of infection, surgical closure of ulcer and missed follow-up appointments during follow-up.

Table S10. Device adherence - ulcer healing.

Reference	Population	Risk of bias	Ulcer outcome	Factor + outcomes	Conclusion
Crews et al. 2016 [5]<br>Prospective cohort study<br>Period: 2008–2012<br>Location: UK and USA (multicenter)	*N*: 79<br>Male: 84%<br>Age: 56.5 ± 9.6 years<br>Type 2: 100%<br>Neuropathy: not presented<br>Ulcer classification: Texas 1A: 72%, Texas 1B: 12%, Texas 2A: 15%, Texas 2B: 1%<br>Location: not presented<br>Area: 23.0± 28.8 cm²	QUIPS: M-L-L-M-M-M<br>21-item score: 10/17	At 6 weeks:<br>Healed: 24% (*n* = 19)	% of activity for which off-loading device worn:<br>Healed: 57 ± 24<br>Non-healed: 59 ± 22	No association (r = −0.043, *p* = 0.705)
				Total adherent activity hours over 6 weeks:<br>Healed: 8126 ± 8161<br>Non-healed: 8216 ± 5566	No association (r = −0.006, *p* = 0.957)
			At 6 weeks:<br>Mean ulcer area: 10.6 ± 15.5 cm²	Mean % of activity for which off-loading device worn: 59 ± 22	Associated (β = −0.16, *p* = 0.038)

Results expressed as mean ± standard deviation. Abbreviations: N = number of participants, type 2 = type 2 diabetes, r = Pearson correlation, β = standardized coefficient: β. Quips domains: (1) Study participation, (2) Study attrition, (3) Prognostic factor measurement, (4) Outcome measurement, (5) Study confounding, (6) Statistical analysis and reporting. L = low, M = moderate and H = high risk of bias.

Table S11. Cumulative plantar tissue stress - ulcer healing.

Reference	Population	Risk of Bias	Ulcer Outcome	Factor + Outcomes	Conclusion
Van Netten et al. 2018 [21]<br>Prospective cohort study<br>Period: 2004–2013<br>Location: the Netherlands and Germany (multicenter)<br>Device: removable knee-high or ankle-high offloading device	*N*: 31<br>Male: 81%<br>Age: 60 ± 12.6 years<br>Type 2: 94%<br>Neuropathy: 100%<br>Ulcer classification: 68% Texas 1A and 32% Texas 2A<br>Location: hallux: 45%, MTP1: 26%, MTP2–5: 29%<br>Area: 1.1 ± 0.9 cm^2	QUIPS: L-L-L-M-M-M<br>21-item score: 13/17	At 12 weeks:<br>Healed: 68% (*n* = 21)	Cumulative plantar tissue stress (2 weeks after baseline) (MPa·s/day)<br>Healed: 155 ± 131<br>Non-healed: 207 ± 215	Not associated (95% CI: (-75; 179), *p* = 0.71, *d* = 0.29)
			At 12 weeks, in the adherent group * (*n* = 27):<br>≥75% ulcer area reduction at 4 weeks: 70% (*n* = 19)<br><75% ulcer area reduction at 4 weeks: 30% (*n* = 8)	Cumulative plantar tissue stress (2 weeks after baseline) (MPa·s/day)<br>≥75% ulcer area reduction at 4 weeks: 140 ± 137<br><75% ulcer area reduction at 4 weeks: 275 ± 209	Not associated (95% CI: (-5; 275), *p* = 0.09, *d* = 0.76)

Results expressed as mean ± standard deviation. Abbreviations: *N* = number of participants, type 2 = type 2 diabetes, MPa s/day = Mega Pascal times seconds per day, d = effect size, CI = 95% confidence interval, OR = odds ratio. Quips domains: (1) Study participation, (2) Study attrition, (3) Prognostic factor measurement, (4) Outcome measurement, (5) Study confounding, (6) Statistical analysis and reporting. L = low, M = moderate and H = high risk of bias. * Patients were classified as adherent when they self-reported to have worn their device >50% of the time while being inside and outside the house at ≥80% of their visits.

References

1. Armstrong, D.G.; Lavery, L.A.; Bushman, T.R. Peak foot pressures influence the healing time of diabetic foot ulcers treated with total contact casts. *J. Rehabil. Res. Dev.* **1998**, *35*, 1–5.
2. Armstrong, D.G.; Lavery, L.A.; Holtz-Neiderer, K.; Mohler, M.J.; Wendel, C.S.; Nixon, B.P.; Boulton, A.J.M.M. Variability in Activity May Precede Diabetic Foot Ulceration. *Diabetes Care* **2004**, *27*, 1980–1984, doi:10.2337/diacare.27.8.1980.
3. Chantelau, E.; Haage, P. An Audit of Cushioned Diabetic Footwear: Relation to Patient Compliance. *Diabet. Med.* **1994**, *11*, 114–116, doi:10.1111/j.1464-5491.1994.tb00240.x.
4. Connor, H.; Mahdi, O.Z. Repetitive ulceration in neuropathic patients. *Diabetes Metab. Res. Rev.* **2004**, *20*, 23–28, doi:10.1002/dmrr.446.
5. Crews, R.T.; Shen, B.J.; Campbell, L.; Lamont, P.J.; Boulton, A.J.M.; Peyrot, M.; Kirsner, R.S.; Vileikyte, L. Role and determinants of Adherence to off-loading in diabetic foot ulcer healing: A prospective investigation. *Diabetes Care* **2016**, *39*, 1371–1377, doi:10.2337/dc15-2373.
6. Deschamps, K.; Matricali, G.A.; Desmet, D.; Roosen, P.; Keijsers, N.; Nobels, F.; Bruyninckx, H.; Staes, F. Efficacy measures associated to a plantar pressure based classification system in diabetic foot medicine. *Gait Posture* **2016**, *49*, 168–175, doi:10.1016/j.gaitpost.2016.07.009.
7. Jarl, G.; Tranberg, R. An innovative sealed shoe to off-load and heal diabetic forefoot ulcers–a feasibility study. *Diabet. Foot Ankle* **2017**, *8*, 1–4, doi:10.1080/2000625X.2017.1348178.
8. Kästenbauer, T.; Sauseng, S.; Sokol, G.; Auinger, M.; Irsigler, K. A prospective study of predictors for foot ulceration in type 2 diabetes. *J. Am. Podiatr. Med. Assoc.* **2001**, *91*, 343–350, doi:10.7547/87507315-91-7-343.
9. Grimm, A.; Kastenbauer, T.; Sauseng, S.; Sokol, G.; Irsigler, K. Progression and distribution of plantar pressure in Type 2 diabetic patients. *Diabetes Nutr. Metab.* **2004**, *17*, 108–113.
10. Lavery, L.A.; Armstrong, D.G.; Wunderlich, R.P.; Tredwell, J.; Boulton, A.J.M. Predictive value of foot pressure assessment as part of a population based diabetes disease management program. *Diabetes Care* **2003**, *26*, 1069–1073, doi:10.2337/diacare.26.4.1069.
11. Ledoux, W.R.; Shofer, J.B.; Cowly, M.S.; Ahroni, J.H.; Cohen, V.; Boyko, E.J. Diabetic foot ulcer incidence in relation to plantar pressure magnitude and measurement location. *J. Diabetes Complicat.* **2013**, *27*, 621–626, doi:10.1093/qjmed/hcl140.

12. Lemaster, J.W.; Reiber, G.E.; Smith, D.G.; Heagerty, P.J.; Wallace, C. Daily weight-bearing activity does not increase the risk of diabetic foot ulcers. *Med. Sci. Sports Exerc.* **2003**, *35*, 1093–1099, doi:10.1249/01.MSS.0000074459.41029.75.
13. Mueller, M.J.; Tuttle, L.J.; Lemaster, J.W.; Strube, M.J.; McGill, J.B.; Hastings, M.K.; Sinacore, D.R. Weight-bearing versus nonweight-bearing exercise for persons with diabetes and peripheral neuropathy: A randomized controlled trial. *Arch. Phys. Med. Rehabil.* **2013**, *94*, 829–838, doi:10.1016/j.apmr.2012.12.015.
14. Murray, H.J.; Young, M.J.; Hollis, S.; Boulton, A.J.M. The Association Between Callus Formation, High Pressures and Neuropathy in Diabetic Foot Ulceration. *Diabet. Med.* **1996**, *13*, 979–982, doi:10.1002/(sici)1096-9136(199611)13:11<979::aid-dia267>3.3.co;2-1.
15. Najafi, B.; Grewal, G.S.; Bharara, M.; Menzies, R.; Talal, T.K.; Armstrong, D.G. Can't Stand the Pressure: The Association between Unprotected Standing, Walking, and Wound Healing in People with Diabetes. *J. Diabetes Sci. Technol.* **2017**, *11*, 657–667, doi:10.1177/1932296816662959.
16. Pham, H.; Armstrong, D.G.; Harvey, C.; Harkless, L.B.; Giurini, J.M.; Veves, A. Screening techniques to identify people at high risk for diabetic foot ulceration: A prospective multicenter trial. *Diabetes Care* **2000**, *23*, 606–611, doi:10.2337/diacare.23.5.606.
17. Caselli, A.; Pham, H.; Giurini, J.M.; Armstrong, D.G.; Veves, A. The forefoot-to-rearfoot plantar pressure ratio is increased in severe diabetic neuropathy and can predict foot ulceration. *Diabetes Care* **2002**, *25*, 1066–1071, doi:10.2337/diacare.25.6.1066.
18. Qiu, X.; Tian, D.H.; Han, C.L.; Chen, W.; Wang, Z.J.; Mu, Z.Y.; Liu, K.Z. Plantar pressure changes and correlating risk factors in Chinese patients with type 2 diabetes: Preliminary 2-year results of a prospective study. *Chin. Med. J. Engl.* **2015**, *128*, 3283–3291, doi:10.4103/0366-6999.171394.
19. Schneider, K.L.; Crews, R.T.; Subramanian, V.; Moxley, E.; Hwang, S.; DiLiberto, F.E.; Aylward, L.; Bean, J.; Yalla, S. Feasibility of a Low-Intensity, Technology-Based Intervention for Increasing Physical Activity in Adults at Risk for a Diabetic Foot Ulcer: A Mixed-Methods Study. *J. Diabetes Sci. Technol.* **2019**, *13*, 857–868, doi:10.1177/1932296818822538.
20. Ulbrecht, J.S.; Hurley, T.; Mauger, D.T.; Cavanagh, P.R. Prevention of recurrent foot ulcers with plantar pressure-based in-shoe orthoses: The CareFUL prevention multicenter randomized controlled trial. *Diabetes Care* **2014**, *37*, 1982–1989, doi:10.2337/dc13-2956.
21. Van Netten, J.J.; van Baal, J.G.; Bril, A.; Wissink, M.; Bus, S.A. An exploratory study on differences in cumulative plantar tissue stress between healing and non-healing plantar neuropathic diabetic foot ulcers. *Clin. Biomech.* **2018**, *53*, 86–92, doi:10.1016/j.clinbiomech.2018.02.012.
22. Waaijman, R.; De Haart, M.; Arts, M.L.J.; Wever, D.; Verlouw, A.J.W.E.; Nollet, F.; Bus, S.A. Risk factors for plantar foot ulcer recurrence in neuropathic diabetic patients. *Diabetes Care* **2014**, *37*, 1697–1705, doi:10.2337/dc13-2470.
23. Crawford, F.; Inkster, M.; Kleijnen, J.; Fahey, T. Predicting foot ulcers in patients with diabetes: A systematic review and meta-analysis. *Q. J. Med.* **2007**, *100*, 65–86, doi:10.1093/qjmed/hcl140.
